# An optimized informer model design for electric vehicle SOC prediction

**DOI:** 10.1371/journal.pone.0314255

**Published:** 2025-03-11

**Authors:** Xin Xie, Feng Huang, Yefeng Long, Youyuan Peng, Wenjuan Zhou

**Affiliations:** Electrical and Information Engineering College, Hunan Institute of Engineering, Xiangtan, Hunan Province, China; Thapar Institute of Engineering and Technology: Thapar Institute of Engineering and Technology (Deemed to be University), INDIA

## Abstract

SOC prediction is of great value to electric vehicle status assessment. Informer model is better than other models in SOC prediction, but there is still a gap in practical application. Therefore, based on the health assessment algorithm, a new optimized Informer model is proposed to predict SOC. Firstly, the health assessment is carried out through the historical running data of the electric vehicle to obtain the health matrix. Then, the health matrix is used to improve Encoder and Decoder modules and improve the prediction accuracy and speed of Informer model. Subsequently, the health matrix is utilized to optimize the prediction logic, reduce the influence of truncation error, and further improve the SOC prediction accuracy. Finally, using the Informer model before and after optimization, SOC prediction is performed using four different datasets. The results indicate that after optimizing the En-De module of Informer, prediction accuracy improved by approximately 15%, with prediction speed increasing by about 100%. Furthermore, optimizing the prediction logic to reduce truncation error further enhanced Informer’s prediction accuracy by around 20%.

## 1 Introduction

State of Charge(SOC) prediction is of great significance to the assessment of electric vehicle status. Because of the influence of SOC on power output, energy management and operation of electric vehicles [1], SOC predictions have become a hot topic in electric vehicle research.

At present, SOC predictions of electric vehicle can be roughly divided into three research directions: SOC prediction through battery working condition; SOC prediction based on the running state change of the electric vehicle; and SOC prediction by combining the historical data of the same type of vehicle on the basis of the two.

Silva et al. [2] analyzed the relationship between the operation of lithium batteries and emissions gases, predicting the remaining SOC percentage based on CO2, NOx, and other gas concentrations in the circulating air inside electric vehicles, but the method needs the support of high-accuracy air data. If the research of Alarrouqi RA et al. [3] is referred to and the low-accuracy air data is used for battery state analysis and SOC prediction, the model prediction accuracy is reduced due to the influence of truncation error; Tekin M et al. [4] estimates the operating parameters of the electric vehicle and predicts SOC through the physical structure change of battery interlayer under different charging voltages. Due to the assumption in this law that transmission delays are fixed, when communication blocks occur and transmission speeds decrease, causing additional latency in data transmission, predictive results will exhibit noise; Kang H C et al. [5] starts with the parameter change of the battery and trains the SOC prediction model through the change of battery parameter by using the phenomenon of thermal expansion and cold contraction of the battery, but the method requires that the temperature of the battery is raised to 400°C and then slowly decreased, and the initial data is difficult to collect in actual situation, causing difficulty in prediction; Mustaffa Z et al. [6] uses the temperature difference of the surface layer of the battery to require a prediction model of surface temperature-SOC from a thermodynamic point of view. However, it is difficult to collect the surface temperature data of a single battery because electric vehicles adopt an integrated battery cluster as a power supply.

Jiang N et al. [7] obtains a prediction model of charging voltage, charging current, total voltage, ambient temperature and the remaining percentage of SOC under different operating conditions under different environmental temperatures. The model exhibits significant prediction errors near the transition between charging and discharging states; Yao Z et al. [8] divided the whole operation interval into several sub-intervals according to the changes of vehicle speed such as acceleration, deceleration and uniform speed, and each sub-interval adopts different SOC prediction strategies to improve the prediction accuracy. When integrating all sub-interval strategies to predict SOC for a new complete operational interval, the prediction accuracy is adversely affected by noise induced by marginal effects and the impact of multi-value predictions, resulting in suboptimal forecasting performance; Sarmokadam S et al. [9] analyzes influence of AC module on SOC and gave the prediction model of SOC according to characteristic data of AC module under different operation conditions. Because the reaction of the mechanical part of the electric vehicle to the AC module is not taken into account, the SOC prediction error of the model is large in the operating range characterized by alternating acceleration and deceleration; Gong et al. [10] analyzed the relationship between electric motor shaft rotation under different operating conditions, vehicle driving speed, and energy consumption of the energy storage module. They proposed a torque-speed-SOC predictive model based on their findings. The model has fewer prediction steps and lower complexity of prediction calculation. The disadvantage of the model is that the prediction accuracy of the model is poor when the running state changes frequently and the torque of the motor changes sharply.

Shao et al. [11] utilized historical data of the same vehicle model at different speeds to derive predictive models for the rate of change of SOC under various operating conditions, indirectly predicting SOC. Although the probability of multi-value prediction is reduced, the influence of SOC characteristic curve deviation is ignored, resulting in the increase of system error; Wang R et al. [12] carry out classified training on the historical data of the same vehicle type and different roads to obtain a speed-SOC prediction model. However, this method assumes that the SOC characteristic curve remains fixed and does not change with varying operational states, leading to poor prediction accuracy of the resulting model; Zhu C et al. [13] using environmental temperature variations, classified historical data for training and obtained predictive models for SOC depletion rates at different temperatures, and based on the model and the SOC margin, the total amount of the SOC at the next moment is predicted. On this basis, Li Q et al. [14] estimate the amount of change in SOC by a weighted average of the rate of change, taking into account the part aging probability of the mechanical part. Both methods refer to the standard SOC characteristic curve to train predictive models, without considering the system errors caused by SOC characteristic curve shifts, resulting in poor prediction accuracy of the model; Lim H et al [15] train the SOC prediction model through the historical data of uniform speed driving at different altitudes, and directly predict the SOC; Guo N et al. [16] train the traction power prediction model by using the historical data of climbing driving under different gravity constants, and indirectly predict the SOC through relevant calculation of mechanics. Due to only considering gravity’s impact on driving speed and not its effect on mechanical components, both methods struggle to handle system errors caused by SOC characteristic curve shifts. As a result, the predictive models obtained exhibit poor robustness against disturbances.

The existing optimization algorithms have addressed some deficiencies in the neural network model but have not significantly enhanced the SOC prediction performance of the model. Selvaraj V et al. [17] combined Bayesian decision mode with Support Vector Regression (SVR), Gaussian Process Regression (GPR) and Linear Regression to improve the SOC multi-value prediction problem of state boundary, but increased the complexity of calculation, and the prediction error of the model was not significantly reduced; Kim D M et al. [18] proposed an improved Deep Neural Network (DNN) for SOC prediction of Fuel Cell Electric Vehicles (FCEVs) based on the changing characteristics of the operating state of FCEVs. The method has fewer prediction steps and higher prediction accuracy, but the prediction calculation is complex, the single prediction time is long, and the hardware requirement is high, which is difficult to be applied in practice; Singla P et al. [19] introduces AND, OR and NOT logic judgment on the original Perturb & Observe (P&O) model to replace the conventional threshold judgment to carry out SOC prediction, although the multi-value prediction frequency is reduced, but the truncation error is not processed in time, and the obtained P&O model exhibits prolonged fixed-value prediction issues when predicting SOC during steady-state operation ranges; Altun Y E et al. [20] applies multi-mode prediction to the existing Artificial Neural Network (ANN). The prediction accuracy of the ANN is temporarily improved, however, when the significant digits after the decimal point of the SOC data are 1-2 bits lower than those of other characteristic data, the prediction accuracy of the ANN is significantly reduced.

As a new prediction model, Informer model has attracted the attention of researchers because of its excellent performance. However, the current Informer model is difficult to meet the high requirements of accuracy and speed for SOC prediction of electric vehicles. Through the health assessment algorithm, the model structure can be improved, the prediction process can be optimized, and the prediction performance of Informer can be improved.

Based on this, a new optimized Informer model for electric vehicle SOC prediction is proposed. Compared to existing SOC prediction models and other Informer models, it features the following characteristics:

1. Structural optimization: Through health assessment, improving the En-De module, optimizing the model structure, and without increasing data costs, enhancing the predictive performance of Informer.
2. Logical Optimization: Addition of fundamental state variables allows for indirect SOC prediction through equivalent conversion, reducing the adverse effects of truncation errors. This further enhances the predictive performance of Informer while retaining the benefits of structural optimization.

This paper is divided into 5 parts: Part 1 focuses on health assessment and optimization analysis, Part 2 analyzes the Informer model and the En-De optimization scheme, Part 3 examines truncation error and proposes prediction logic optimization, Part 4 involves model testing and result analysis, and Part 5 presents the conclusions.

## 2 Health assessment

Health assessment is a weight optimization algorithm proposed by Beatrice Greaves, Lars Landberg et al. 2011 [21]. It transforms the correlation weights between features into a health matrix based on the statistical probability distribution characteristics of model outputs.

**Fig 1:**
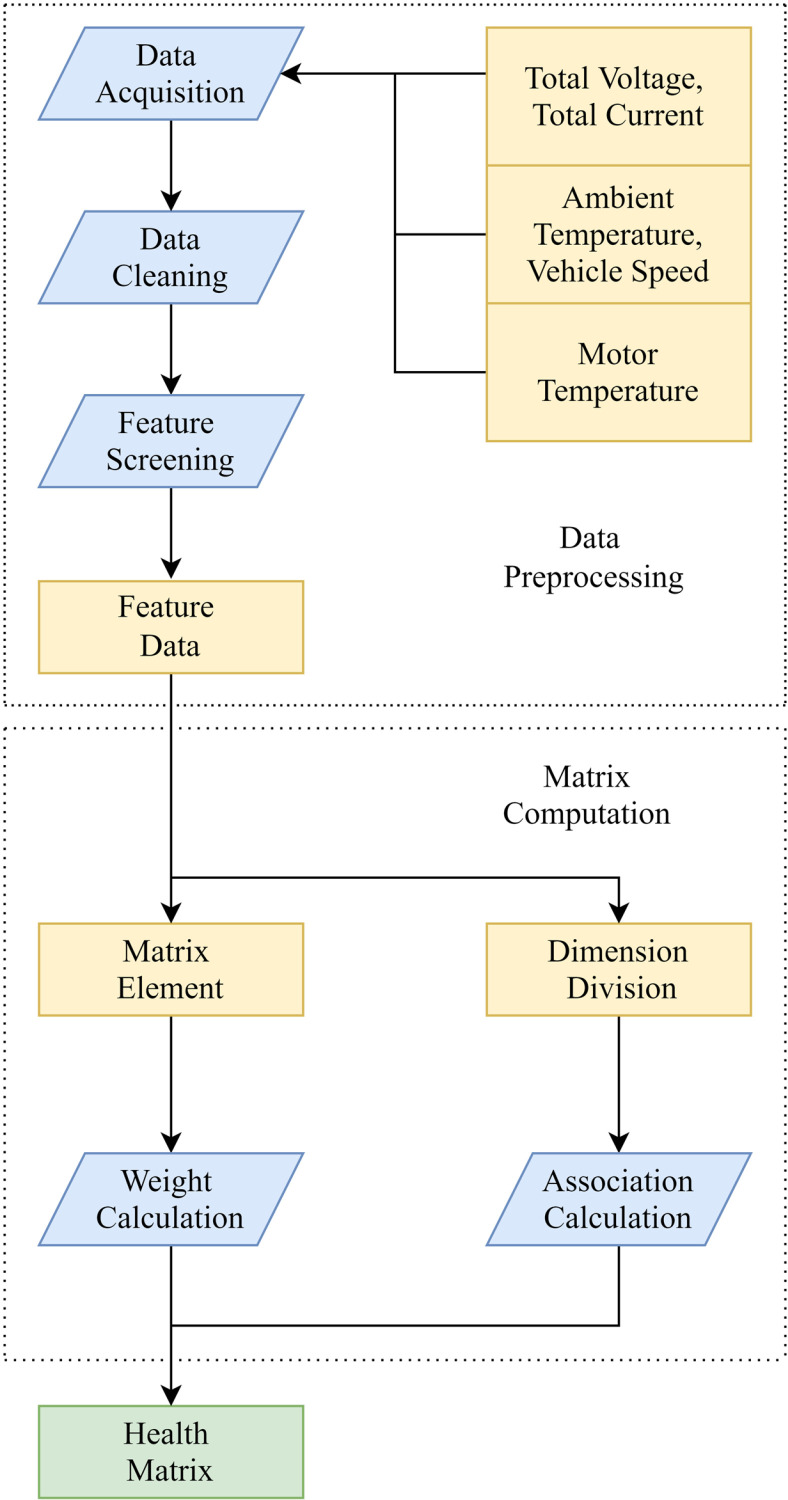
Health assessment process diagram.

The health assessment process is shown in Fig 1:

(1) Data Acquisition: The operational data of total voltage, total current, ambient temperature, vehicle speed, motor temperature, and similar parameters is acquired through the data acquisition module [22];
(2) Data Cleaning: Eliminate null value, abnormal value and noise fluctuation of operation data to improve data reliability [23];
(3) Feature Screening: Feature data is screened out from the cleaned operation data according to the strength of SOC Correlation [24];
(4) Matrix Computation: Using feature data, after determining matrix elements and dimension division schemes, matrix calculations are conducted. This includes computing the weights of each feature’s impact on SOC under different operating conditions, as well as assessing the dimensions of interactions among different features. [25];

The brief calculation formula for Matrix Computation is as follows:


$$f\left(x;\mu ,\Sigma \right)=\frac{1}{{\left(2\pi \right)}^{\frac{i}{2}}{\left|\Sigma \right|}^{\frac{1}{2}}}exp\left[\frac{1}{2}{\left(x-\mu \right)}^{T}\Sigma^{-1}\left(x-\mu \right)\right]$$ (1)

where *i* is the feature number from 1 to *N*, *μ* is the mean vector, and *Σ* is the covariance matrix.

(5) Matrix Generation: The calculation results of matrix are arranged sequentially to obtain the weight matrix $Q_{N\times 1}$. Each element of *Q*, representing a different operating state, specifies a weight factor for the feature [26]. Factor analysis was then used on the running data to generate the correlation matrix $R_{1\times N}$. Finally, *Q* is multiplied by *R* to obtain the health matrix *A*, namely:

$$A_{N\times N}=Q_{N\times 1}R_{1\times N}$$ (2)

where *N* is the dimension of the matrix, usually 4-12. When *N* is greater than 12, dimension reduction of operation data is required.

The optimization analysis of health assessment is as follows

Let the first layer input of the prediction model $z^{\left(0\right)}$, with a principal diagonal element of 1, and a coefficient matrix of $D_{N\times N}^{0}$ between (0,1) of the remaining elements. Sort $D^{0}$ so that the order of $D^{0}$ row and column labels is consistent.

During the prediction process, the coefficient matrix $D^{i}$ for the *i* - th layer input $z^{\left(i-1\right)}$ can be viewed as the *i* - th power operation of $D^{0}$:


$$D^{i}={\left[D^{0}\right]}^{i}=D^{0}\times D^{0}\times \cdots \times D^{0}$$ (3)

The health assessment optimization can be regarded as multiplying the input $z^{\left(0\right)}$ with the health matrix *A* to obtain $z_{1}^{\left(0\right)}$, that is, $z_{1}^{\left(0\right)}=z^{\left(0\right)}\times A$, then the coefficient matrix of $z_{1}^{\left(0\right)}$ is $D_{1}^{0}$, which is calculated as follows:


$$D_{1}^{0}=D^{0}\times A$$ (4)

In Equation (4), *A* reduces the weakly correlated weight of $D^{0}$, that is, on the basis of maintaining the dominance and symmetry of the dominant diagonal, the weakly correlated principal element of $D^{0}$ is adjusted to between (0,1). After optimization, all $D^{0}$ in Equation (3) is replaced by $D_{1}^{0}$, and the weight of weak correlation decreases every time the forward calculation of the model is performed, so as to reduce the influence of weak correlation features and improve the prediction performance of the model.

The effects of input *z* and coefficient matrix *D* are different due to different prediction processes, and the prediction performance of the model is improved differently after optimization.

The health assessment algorithm, when applied to the SOC prediction of electric vehicles, has two main drawbacks:

1. High Data Costs: High dimensionality of operation data required by the algorithm leads to large workload of data collection, data cleaning and feature screening, and increases data costs [27];
2. High Complexity: In order to select the main features, a high-order selection module is introduced, which increases the algorithm complexity [28];

In order to solve the above problems, reduce the algorithm complexity and reduce the data costs, the following measures can be taken:

Firstly, the principal component analysis method is used to simplify the high-order selection module and reduce the complexity of the algorithm.

Then, the data costs are reduced by dimensionality reduction and decoupling of the running data.

## 3 Informer prediction model

### 3.1 Model principle

Informer is a self-attention prediction model proposed by Zhou Haoyi et al. in 2021 [29]. Different from other prediction models, Informer adopts the structure of encoder-decoder separation. In the prediction process, there is no other connection between encoder and decoder except the transmission of coding output, and there is no feedback mechanism in all prediction steps.

**Fig 2:**
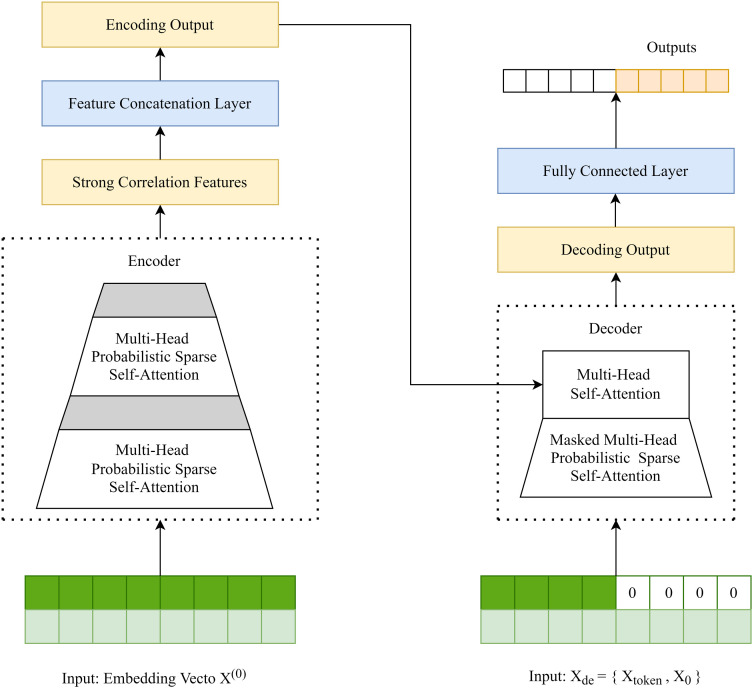
Principle diagram of the informer model.

The principle diagram of Informer model is shown in Fig 2.

In Informer, there are three key structures:

(1) Self-Attention Distilling Operation: The encoder is composed of a plurality of encoding layers, each encoding layer uses sparse sampling, reduces the sequence length, and outputs strong correlation features in the last layer, and then obtains the encoded output through the feature connection layer.
(2) Generative Style Decoder: Decoder’s decoding operation is carried out based on masking multi-head attention, and decoding output with a specified length can be obtained only by forward calculation, so that the occurrence probability of multi-value prediction is reduced while cumulative error diffusion is prevented.
(3) Prob Sparse Self-Attention Mechanism: Based on the conclusion that a small part of dot product focuses on contribution, Informer improves the attention degree of strong correlation features, and improves the self-attention mechanism by increasing the weight of strong correlation features in the prediction process.

The existing Informer model has the following shortcomings:

1. Frequent Calculation: In order to improve the attention of strong correlation features, the Encoder module needs to perform two dot product calculations, which reduces the prediction speed of Informer [30–31];
2. Decoding Defect: Decoding input, consisting of 0 placeholder and encoded output. The weight of the placeholder is too high, which affects the decoding reliability of the Decoder module and reduces the prediction accuracy of Informer [32–33].
3. High Complexity: Sparse sampling query of self-attention mechanism increases model complexity and reduces the lightweight advantage of Informer [34–35].

In order to make up for the above shortcomings and improve the prediction performance of Informer, it is planned to obtain the health matrix through health assessment, and optimize the model structure of Informer from two aspects of encoder and decoder.

### 3.2 Encoder optimization

Encoder has multiple encoding layers, each with a self-attention sublayer and a convolutional sublayer. Let the input of the *i* - th encoding layer be $X^{\left(i-1\right)}$ and the output be $X^{\left(i\right)}$. In each encoding layer, $X^{\left(i-1\right)}$ is advanced forward by multi-head self-attention, and then convolutional calculations are used to reduce the sequence length to obtain $X^{\left(i\right)}$, i.e.,:


$$X^{\left(i\right)}=Conv\left(Attention\left(X^{\left(i-1\right)}\right)\right)$$ (5)

In Equation (5), *Conv* represents convolutional calculation, *Attention* represents probabilistic sparse self-attention, and after the *Attention* $\left(X^{\left(i-1\right)}\right)$ operation, the output *p* of the self-attention sublayer can be obtained, that is:


$$p=softmax\left(\frac{\bar{Q}K^{T}}{\sqrt{d_{k}}}\right)V$$ (6)

In Equation (6), *softmax* is the normalization function, $\bar{Q}$ is the probability sparse matrix obtained after sampling $X^{\left(i-1\right)}$, $\sqrt{d_{k}}$ is the variance, *K*, $K^{T}$,*V* is the adjustment matrix of the sampling key value, and *p* is the output of the self-attention sublayer.

In $X^{\left(i-1\right)}$, the weights of all features are equal, and the weights of the strongly correlated features in $X^{\left(i-1\right)}$ are increased through the health matrix *A*, that is:


$$X_{1}^{\left(i-1\right)}=X^{\left(i-1\right)}\times A$$ (7)

In Equation (5), $X_{1}^{\left(i-1\right)}$ is used instead of $X^{\left(i-1\right)}$, which can accelerate the generation of sparse sampling and $\bar{Q}$, and improve the encoding speed of Encoder and the prediction speed of Informer.

The optimization analysis of Encoder is as follows

In Equation (6), the *i* - th query element of $\bar{Q}$, which is obtained by the following operation:


$$pq_{i},K,V=\underset{j}{\sum }\frac{k\left(q_{i},k_{j}\right)}{\sum_{l}k\left(q_{i},k_{l}\right)}v_{j}=Exp_{p\left(k_{j}\text{|}q_{i}\right)}\left[v_{j}\right]$$ (8)

where $q_{i}$, $k_{i}$, $v_{i}$ represent the *i* - th line in $\bar{Q}$, *K, V*, $p\left(k_{j}\text{|}q_{i}\right)=k\left(q_{i},k_{j}\right)/\underset{l}{\sum }k\left(q_{i},k_{l}\right)$, $k\left(q_{i},k_{j}\right)$ represents the asymmetric exponential kernel *Exp* $q_{i}k_{j}^{T}$ / $\sqrt{d}$), and $\sqrt{d}$ represents the variance.

In Equation (6) and Equation (8), the *Top-u* query with a sparse metric *M*($q_{i}$,*K*) is used, i.e.,:


$$Mq_{i},K=\underset{j}{\max}\left\{\frac{q_{i}k_{j}^{T}}{\sqrt{d}}\right\}-\frac{1}{L_{K}}\overset{L_{K}}{\underset{j=1}{\sum }}\frac{q_{i}k_{j}^{T}}{\sqrt{d}}$$ (9)

where $L_{K}$ denotes the sequence length of matrix *K*.

In sparse self-attention, the length of the query and the key are equal, i.e., $L_{Q}$ =  $L_{K}$ = *L*, and the computational complexity is *O*(*L* · *ln L*). Since all features of $X^{\left(i-1\right)}$ have equal weights, in order to increase the weights of strongly correlated features, *Top-u* queries need to perform two dot product calculations, which affects the encoding speed of Encoder.

By replacing $X_{1}^{\left(i-1\right)}$ with $X^{\left(i-1\right)}$ as the input of each encoding layer, the weight of strongly correlated features can be increased, and the *Top-u* query only needs to perform one dot product calculation, and the computational complexity of sparse self-attention is reduced from*O*(*L* · *ln L*) to *O*($\frac{L}{2}\cdot$ *· ln L*), which improves the encoding speed and accelerates theprediction speed of the Informer model.

### 3.3 Decoder optimization

Let the Decoder be composed of *L* decoding sublayers overlapping. In the decoding process, initial input $c_{0}$ and feature data *y* need to undergo *L* decoding operations to obtain the decoding output $c_{L}$, that is:


$$c_{L}=Decoder\left(y,c_{L-1}\right)=Decoder_{L}\left(Decoder_{L-1}\left(\cdot \cdot \cdot Decoder_{1}\left(y,c_{0}\right)\cdot \cdot \cdot \right)\right)$$ (10)

where $Decoder_{i}\left(\cdot \right)$ represents the decoding computation of the *i* - th decoding sublayer, and 0≤i≤L. $c_{0}$ is the input of the first decoding sublayer, $c_{i-1}$ is the input of the *i* - th decoding sublayer, and *y* represents the characteristic data at the prediction time.

In $c_{i-1}$, all features are weighted equally. The weight of the strongly correlated features in the $c_{i-1}$ can be increased through the health assessment, namely:


$$c_{L}=Decoder\left(y,c_{L-1}\right)=Decoder_{L}\left(Decoder_{L-1}\left(\cdot \cdot \cdot \left(Decoder_{1}\left(y,c_{0}\right)\cdot h\right)\cdot \cdot \cdot \right)\right)$$ (11)

where *h* is the health factor, which is obtained by the health matrix $A_{N\times N}$ compression transformation. In each decoding sublayer, the decoding output $x_{i}$ obtained by $\left(Decoder_{i}\left(y,c_{0}\right)\right)$ is multiplied by the health factor *h* and used as the input of the next decoding sublayer, which can improve the weight of strongly correlated features, and the decoding accuracy of Decoder and the prediction accuracy of Informer model can be increased.

The optimization analysis of Decoder is as follows

The input $c_{0}$ of the first decoding sublayer can be expressed as a sequence of $X_{de}^{t}$, i.e.,:


$$X_{de}^{t}=Concat\left(X_{token}^{t},X_{0}^{t}\right)\in R^{\left(L_{token}+L_{y}\right)\times d_{model}}$$ (12)

where $X_{token}^{t}\in R^{L_{token}\times d_{model}}$ is the encoded output in Fig 2, $X_{0}^{t}\in R^{L_{y}\times d_{model}}$ is the sequence placeholder with a scalar of 0 (corresponding to all 0s of the $X_{de}$ in Fig 2), $L_{token}$ represents the sequence length of the encoded output, $L_{y}$ represents the sequence length of the placeholder, $d_{model}$ represents the feature dimension, and *Concat* represents the vector connection. By setting the 0 placeholder and −∞ masking dot product, masked multi-head probabilistic sparse self-attention can be applied to the decoding process of Decoder.

Although the masking dot product can be set to prevent the autoregressive phenomenon of each feature focusing on its own next moment in $X_{de}^{t}$, the weight of the coded output $X_{token}^{t}$ and the placeholder $X_{0}^{t}$ is equal, and the cumulative error caused by $X_{0}^{t}$ spreads every time the forward computation of the decoded sublayer is performed. The diffusion of accumulated errors reduces the decoding accuracy of the Decoder.

The optimization scheme of Equation (11) is equivalent to increasing the weight of $X_{token}^{t}$ and decreasing the weight of $X_{0}^{t}$, and it is known from Equation (3) and Equation (4) that the cumulative error caused by $X_{0}^{t}$ is reduced once every time the forward decoding is carried out, which improves the decoding reliability of Decoder and thus improves the prediction accuracy of Informer.

### 3.4 En-De optimization

Based on Section 2.1, Informer adopts a structure with separated Encoder and Decoder, and there are no feedback mechanisms. According to Section 2.2 and Section 2.3, optimizing the Encoder module can improve the Informer prediction speed, and optimizing the Decoder module can improve the Informer prediction accuracy, and the two optimization effects do not conflict. En-De combination optimization can simultaneously improve the prediction speed and accuracy of Informer.

## 4 Prediction logic optimization

### 4.1 Truncation error

In Literatures [3], [19] and [20], there is a problem that truncation errors cause a decrease in prediction accuracy, which frequently occurs in certain cases. In fact, all prediction models have truncation errors. The reason is that, due to the accuracy limitation of the acquisition module, the significant digit of SOC is only 0 or 1 digit after the decimal point in most cases, which is much lower than the significant digit of 3-4 digits after the decimal point of other characteristics. During prediction, the model standardizes data precision by aligning the significant figures of predicted values with the feature in the training data that has the highest number of significant figures. The larger the significant bit difference between the predicted value and the true value, the greater the SOC truncation error.

Assuming that the predicted value of SOC is 28.5273 for a certain time, when the true value is recorded as 28.5, the truncation error is {(28.5273-28.5)/ 28.5273} × 100% =  0.096%, and when the true value is recorded as 28, the truncation error is {(28.5273-28)/ 28.5273} × 100% =  1.848%, the truncation error is increased by about 20 times. From the analysis in Sections 1 and 2.3, the truncation error will accumulate with the forward calculation. When the truncation error accumulates to a certain extent, the prediction accuracy of the model will decrease significantly. In addition to the limitation of acquisition accuracy, the state change of the power supply module leads to the virtual electricity phenomenon that the actual value of SOC is lower than the measured value [36–37], and the long-term low-speed operation of electric vehicles, with minimal state changes, leads to the phenomenon of fixed SOC measurement [38–39], which will result in the truncation error.

In order to reduce the negative influence of truncation error, the basic health matrix can be obtained through health assessment, basic state variables are added, equivalent conversion is carried out, SOC indirect prediction is used instead of SOC direct prediction, and the prediction logic of Informer is optimized.

### 4.2 Prediction logic optimization scheme

Informer’s prediction logic optimization scheme is based on the following four assumptions:

1. Each motor temperature corresponds to a unique SOC characteristic curve. Within the normal operating range, this curve is a smooth and continuous single-value curve. Moreover, the characteristic curves for the same vehicle model at the same temperature are completely consistent. [40–41];
2. All characteristics are smooth and continuous in the normal operation interval and are affected by SOC, motor temperature and other characteristics. Furthermore, when any one feature changes independently while all other characteristics remain unchanged, the rate of change of SOC will correspondingly alter [42–43];
3. In the normal operation range, the SOC characteristic curve of the same vehicle type and different temperature has the same curve trend, and there is only horizontal and vertical translation, resulting in a numerical difference [44–45];
4. The influence of a single feature on SOC is calculated by approximate simplification, i.e., k=m*Δx+n*Δx+l. Among them, *k* is the characteristic influence multiplier and *m* is the influence coefficient of the motor temperature, which is determined by the mechanical structure of the electric vehicle; *n* is the influence coefficient of other features, which is determined by the physical structure of the energy storage module; *∆ x* is the amount of change for the selected feature, and *l* is a constant. Within the normal operating interval, *m*, *n*, and *l* have a corresponding fixed value for each feature, and this value does not change due to the deviation of the SOC characteristic curve [46–47].

The prediction logic optimization scheme based on the above assumptions is as follows:

1. Set the temperature of a certain motor and the corresponding characteristic parameters of the electric vehicle to the basic state by referring to the equipment manual or rated parameters;
2. Using the Informer model optimized with historical data and structural adjustments for prediction, and obtaining the baseline health matrix of the system. Through the matrix elements of this baseline health matrix, we calculate the basic rate of change $v_{0}$ of SOC, as well as the values *m*, *n*, and *l* corresponding to each feature assumed in hypothesis 4.
3. According to the temperature of the motor, the complete normal operating range is divided into several sub-intervals. For the boundary temperatures of the entire range and the intermediate temperatures at the intersections of the sub-intervals, predictions are made using the dataset from step 2 and the optimized Informer model to obtain the characteristic parameters corresponding to each motor temperature when the SOC change rate is $v_{0}$. These characteristic parameters, along with the SOC basic change rate and the corresponding motor temperatures, are labeled as sub-basic states;
4. When predicting, first, we should obtain a predicted value of the motor temperature through an Informer model, find a corresponding basic state or a sub-basic state according to the predicted temperature and then carry out equivalent conversion to obtain a corresponding SOC change rate at the predicted time;

If there are *p* features, the influence rate of the features is calculated to be $k_{1}$, $k_{2}$,···, $k_{p}$. Relative to the basic rate $v_{0}$, the multiple of the prediction rate $v_{1}$ is $k_{0}$, and the relevant calculation equation is as follows:


$$k_{0}=k_{1}*k_{2}*\cdot \cdot \cdot *k_{p}$$ (13)

When it is to be predicted, the corresponding calculation equation for the SOC prediction rate $v_{1}$ is:


$$v_{1}=v_{0}*k_{0}$$ (14)

where $v_{0}$ is the basic rate of change of the SOC, which is obtained in step 2.

1. Combined with the historical value of SOC and the sampling interval, the predicted value of SOC is calculated through the $v_{1}$ of the change rate.

**Fig 3:**
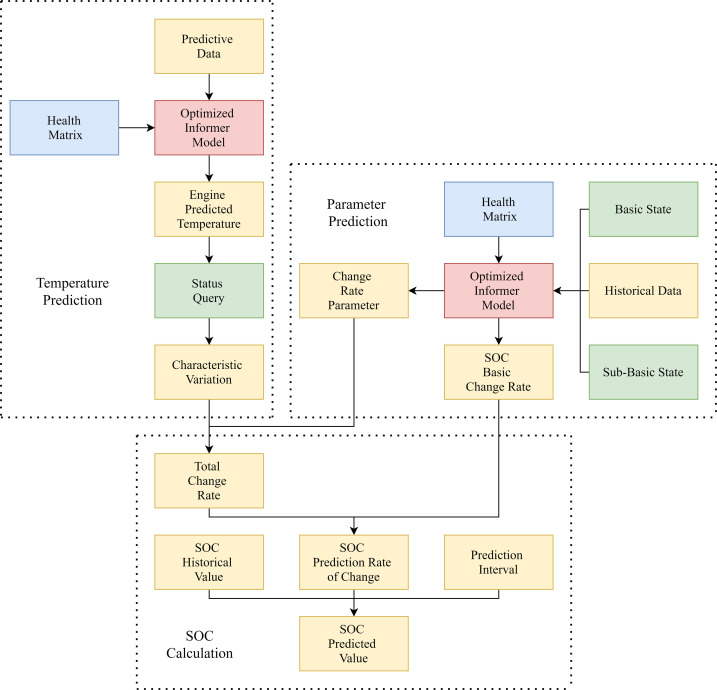
Prediction logic optimization diagram.

The predicted logic optimization diagram is shown in Fig 3. Due to the fundamental health matrix, which is independent of the Informer model and specifically used for the variable matrix of SOC basic states, to avoid misunderstanding, Fig 3 directly labels the SOC basic change rates and the parameters for change rate. The basic health matrix involved in both is not separately labeled.

## 5 Model test

### 5.1 Evaluation indicator

R^2^ (Coefficient of Determination), MSE (Mean Squared Error) and MAE (Mean Absolute Error) are adopted as three evaluation indicators, and the calculation of each indicator is shown in Equation (15)–(17):


$$R^{2}=1-\frac{\sum_{i=1}^{n}{\left(\hat{y_{i}}-y_{i}\right)}^{2}}{\sum_{i=1}^{n}{\left(\bar{y_{i}}-y_{i}\right)}^{2}}$$ (15)

$$MSE=\frac{1}{n}\overset{n}{\underset{i=1}{\sum }}{\left(\hat{y_{i}}-y_{i}\right)}^{2}$$ (16)

$$MAE=\frac{1}{n}\overset{n}{\underset{i=1}{\sum }}\left|\bar{y_{i}}-y_{i}\right|$$ (17)

where $y_{i}$ is the true value, $\hat{y_{i}}$ is the predicted value and $\bar{y_{i}}$ is the true average value. R^2^ indicates the degree of fit, and the closer the value is to 1, the better the fitting effect of the model. MSE, MAE represents the degree of dispersion, and the closer the value is to 0, the smaller the prediction fluctuation of the model.

The prediction accuracy is improved, and the judgment is performed by R^2^ promotion. When MSE and MAE are unchanged or decreased, R^2^ is increased from 0.8 to 0.85 and prediction accuracy is improved by 25%. R^2^ improved from 0.9 to 0.95 with a 50% improvement in prediction accuracy. The improvement in prediction speed is assessed by reducing the predicted time required. When MSE, MAE and R^2^ remain unchanged or decrease, for single prediction, it takes 30s before optimization and 20s after optimization, and the prediction speed increases by 50%; it takes 30s before optimization and takes 10s after optimization, and the prediction speed increases by 200%.

If the optimization is effective, MSE and MAE will also decrease while R^2^ increases. However, due to the different calculation methods of the model, there is a difference in the degree of predicted fluctuation[48] and in order to increase the overall fit, the decrease of the fit of specific points will increase the cost of accident treatment[49]. Therefore, when two indicators improve by more than 10%, and the remaining one decreases by no more than 2%, it is also considered effective optimization.

### 5.2 Simulation environment

The SOC prediction test was performed using 4 different published data sets, each of which is outlined below:

1. Data set of Charging of Chinese Urban Electric Vehicle: This dataset is sourced from the public dataset provided by the 4th Jiangsu Big Data Development and Application Competition—New Energy Track of “SEED” in 2023, including current, voltage, SOC and energy recorded in electric vehicle charging piles in Beijing, Shanghai and Shenzhen, totally 4 characteristics. Beijing data set with No. 1 and Shanghai data set with No. 2 are selected for simulation test;
2. Data set of Battery Module Charging and Discharging of 20 New Energy Vehicles: This data set is sourced from the public data set provided in Literature [50], including the charging and discharging data of 20 electric vehicles, and the data of each electric vehicle includes 9 characteristics including voltage, current, temperature, energy, SOC, etc. Select the running data of No. 2 vehicle and No. 17 vehicle for simulation test;
3. Data set of Power Battery Health of New Energy Vehicles: This data set is sourced from the public data set provided by the 2nd session of Pazhou Algorithm Competition in Guangzhou, including the charging and discharging data of two electric vehicles, and the operation data of each electric vehicle, including such characteristics as speed, voltage, current and SOC. The operation data of two electric vehicles were simulated and tested respectively.
4. Data set of Health Degree of New Energy Vehicles in Guangdong-Hong Kong-Macao Bay Area: This data set is sourced from the public data set provided in the 2022 New Energy Smart Vehicle Big Data Innovation Competition in Guangdong-Hong Kong-Macao Bay Area, including the operation data of five new energy vehicles of each type. The operation conditions of the data set are complex, including not only the charging and discharging conditions, but also the high-low speed operation, sudden braking, continuous acceleration or deceleration, turning and turning and other operation conditions. The operational data of each electric vehicle includes multiple features such as SOC, speed, voltage, current, and temperature. This study selects 12 features, including SOC, for simulation testing.

In the phase of structural optimization, four models including before optimization, Encoder optimization, Decoder optimization and En-De optimization were selected for simulation test. In the logic optimization stage, 4 sets of models are optimized for prediction logic in turn, and 8 sets of models before and after logic optimization are used for simulation test respectively.

From datasets 1 to 4, several subsets of data are partitioned for simulation testing. Each subset contains approximately 20,000 operational records, with the first 80% of the data serving as the training set and the remaining 20% as the test set. All data in datasets 1 to 4 are sourced from real-time data collected by the remote monitoring module of electric vehicles.

In the prediction effect charts, V1 represents non optimization, V2 represents Encoder optimization, V3 represents Decoder optimization, V4 represents En-De optimization, true represents true value. The horizontal axis represents the sequence number of the data; the vertical axis represents the standardized predicted values, with the vertical axis labels capable of taking any real number. Each prediction effect chart is accompanied by a corresponding table that records relevant prediction results.

The simulation test platform is Intel Core i7-7700HQ CPU, 8GB DDR4 RAM, 1TB WDC HDD, and the operating system is Windows 10.

To reduce the impact of dimensional differences, all predictions were normalized as follows:


$$z_{1}=\frac{1}{z_{s}}\left(z_{0}-\bar{z}\right)$$ (18)

where $z_{0}$ represents the unnormalized prediction; $z_{1}$ represents the normalized forecast; $z_{s}$ represents the variance of the forecast and $\bar{z}$ represents the average value of all forecasts.

See Table 1 for Informer Model Parameters.

**Table 1:** Informer model parameters.

	Encoder Modules
**Input**	1x3 Convolution Layer (Step 1, Kernel Width 3)
	Embedded Vector (Dimension *d* = 512)
**Probabilistic Sparse Self-Attention Block**	Multi-Head Probabilistic Sparse Self-Attention (Number of Heads *h* = 16, Dimension *d* = 32), Rejection Rate *p* = 0.1
	Probabilistic Sparse Network (Dimension *d* = 2048), Activation Function is GELU Function, Rejection Rate *p* = 0.1
**Distillation**	1 × 3 Convolution Layer (Step 1, Kernel Width 3), Activation Function is ELU Function
	Max Pooling (Stride = 2)
**Input**	**Decoder Modules**
	1x3 Convolution Layer (Step 1, Kernel Width 3)
	Initial Input (Dimension *d* = 512)
**Reasonable Cover-Up**	Mask Attention Block
**Self-Attention Block**	Multi-Head Self-Attention (Number of Heads *h* = 8, Dimension *d* = 64), Rejection Rate *p* = 0.1
	Probabilistic Sparse network (Dimension *d* = 2048), Activation Function is GELU Function, Rejection Rate *p* = 0.1

### 5.3 Model optimization verification

**Fig 4:**
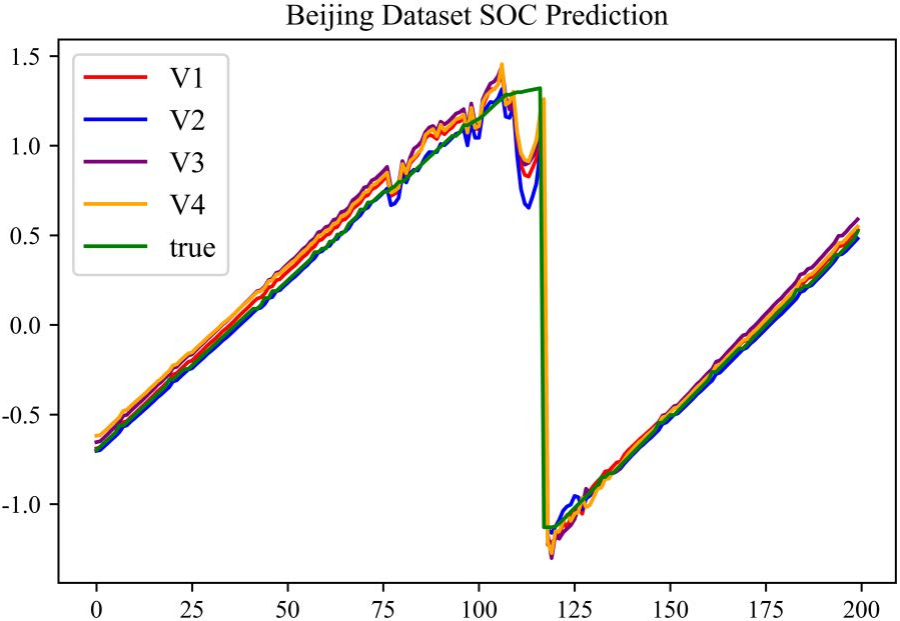
SOC prediction chart for Beijing dataset.

**Fig 5:**
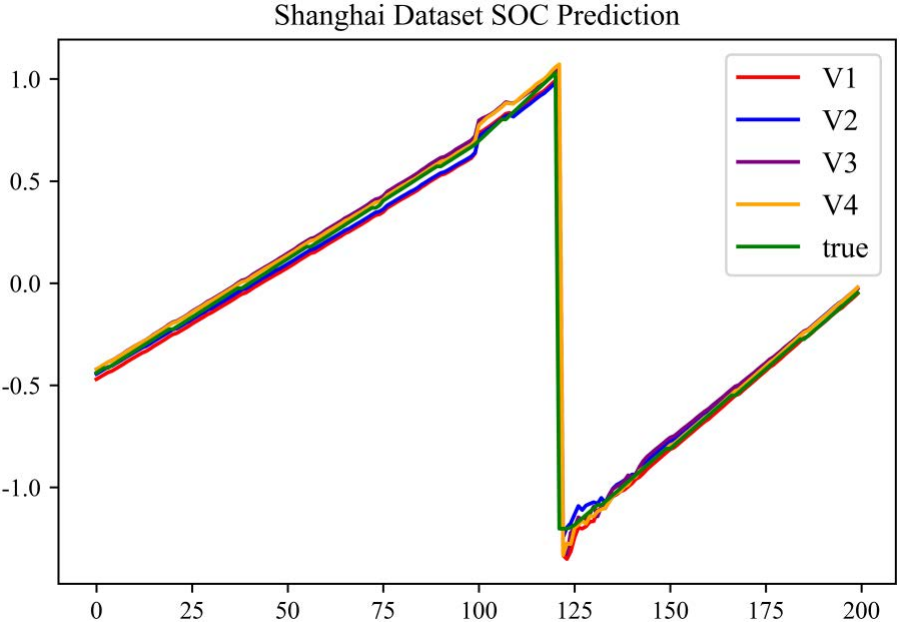
SOC prediction chart for Shanghai dataset.

The prediction results using dataset 1 are shown in Figs 4 and 5.

**Fig 6:**
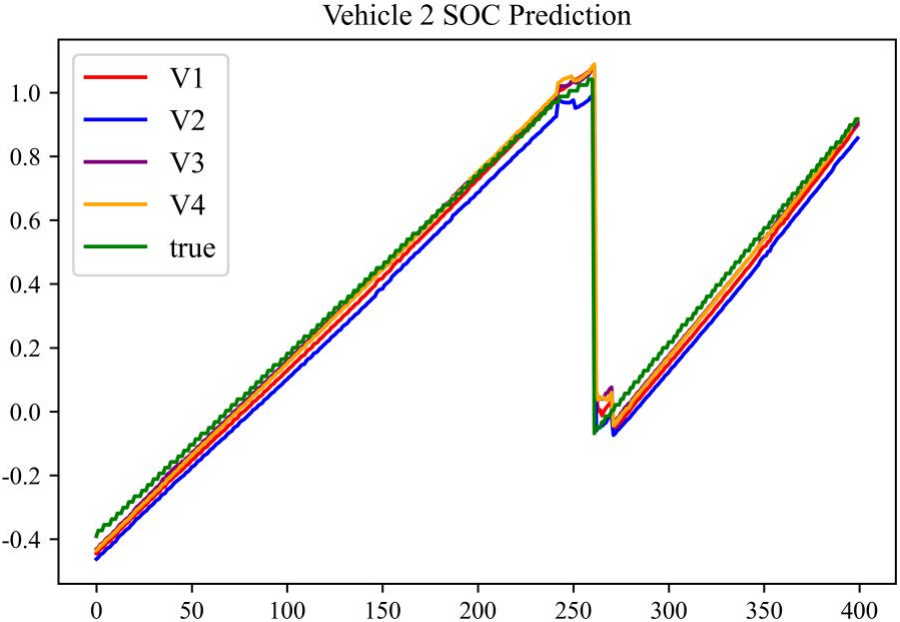
SOC prediction chart for No. 2 vehicle.

**Fig 7:**
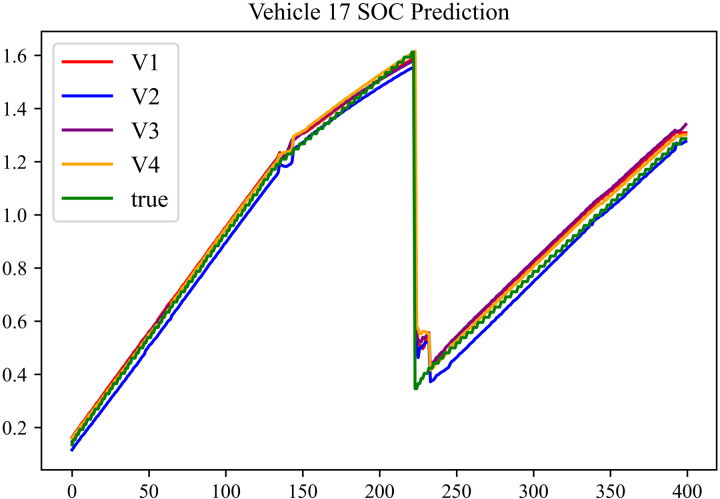
SOC prediction chart for No. 17 vehicle.

The prediction results using dataset 2 are depicted in Figs 6 and 7.

**Fig 8:**
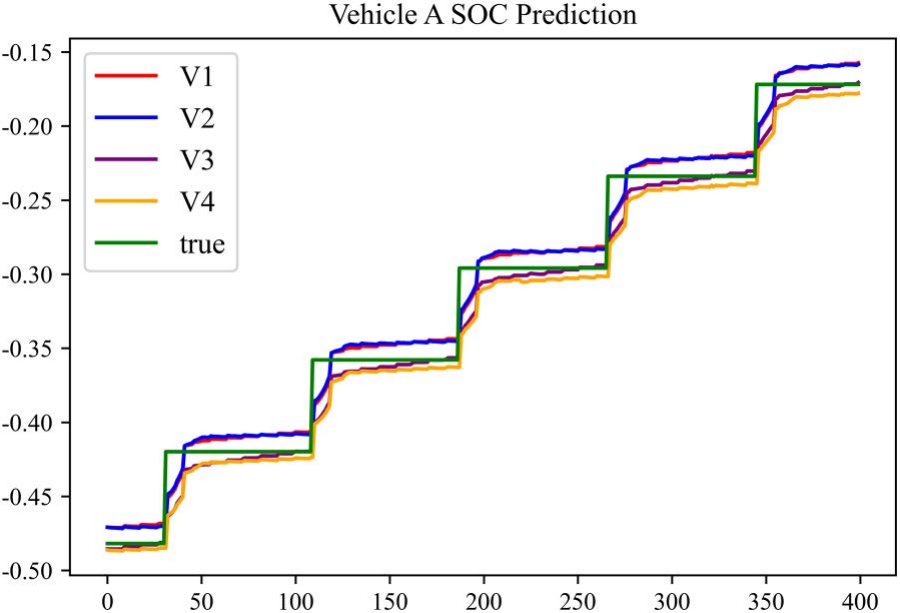
SOC prediction chart for Type A vehicle.

**Fig 9:**
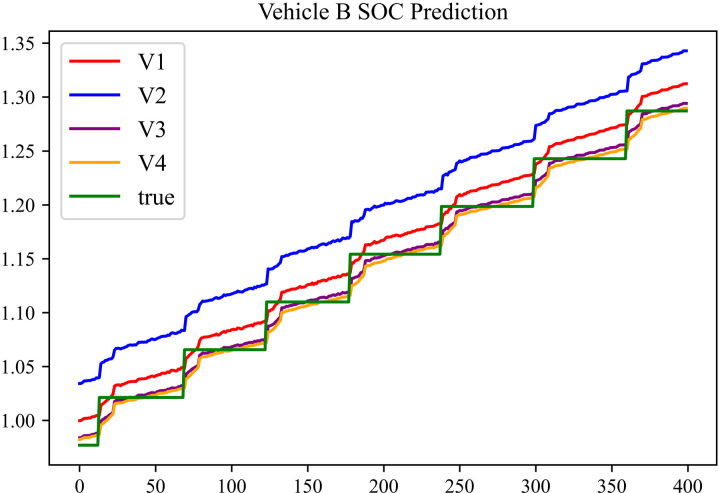
SOC prediction chart for Type B vehicle.

The prediction results using dataset 3 are shown in Figs 8 and 9.

**Fig 10:**
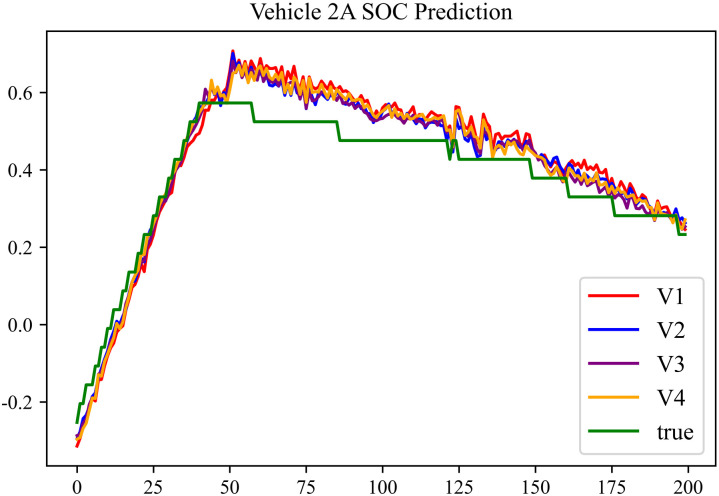
SOC prediction chart for No. 2 Type A vehicle.

**Fig 11:**
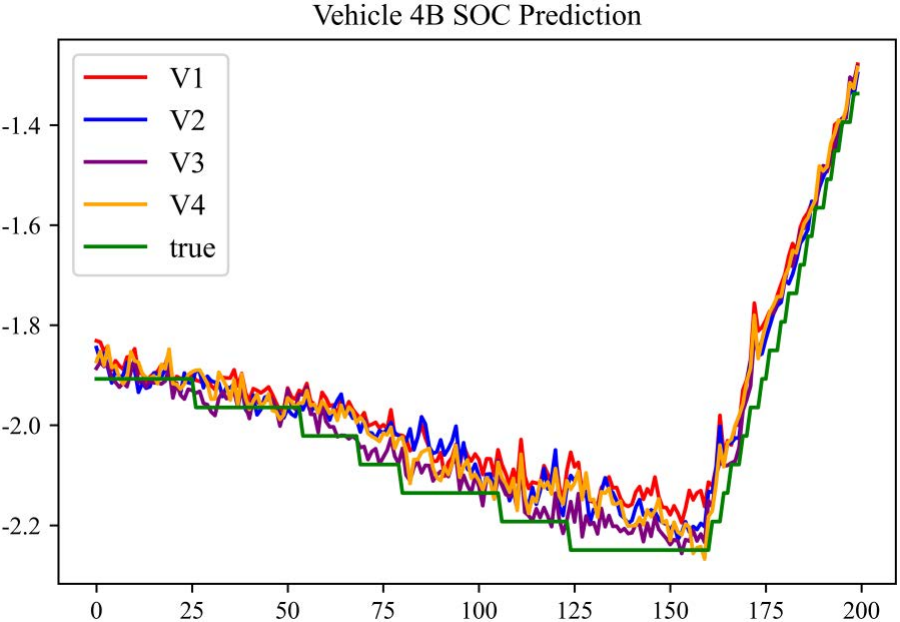
SOC prediction chart for No. 17 Type A vehicle.

The prediction results using dataset 4 are depicted in Figs 10 and 11.

The prediction results of each simulation test are presented in Tables 2–9.

**Table 2:** SOC prediction results for Beijing dataset.

	MSE	MAE	R^2^	Time/ s
**Unoptimized**	0.0689	0.1080	0.9537	235.2762
**En Optimized**	0.0696	0.1093	0.9525	112.8759
**De Optimized**	0.0547	0.0812	0.9630	236.5538
**E-D Optimized**	0.0563	0.0835	0.9615	112.0329

**Table 3:** SOC prediction results for shanghai dataset.

	MSE	MAE	R^2^	Time/ s
**Unoptimized**	0.0235	0.0402	0.9604	238.1326
**En Optimized**	0.0238	0.0404	0.9599	118.4991
**De Optimized**	0.0206	0.0297	0.9666	238.7069
**E-D Optimized**	0.0210	0.0304	0.9662	118.7696

**Table 4:** SOC prediction results for No. 2 vehicle.

	MSE	MAE	R^2^	Time/ s
**Unoptimized**	0.0352	0.0457	0.9589	239.0797
**En Optimized**	0.0357	0.0464	0.9567	117.0568
**De Optimized**	0.0302	0.0311	0.9672	238.9248
**E-D Optimized**	0.0308	0.0315	0.9666	117.6935

**Table 5:** SOC prediction results for No. 17 vehicle.

	MSE	MAE	R^2^	Time/ s
**Unoptimized**	0.0379	0.0451	0.9629	237.7536
**En Optimized**	0.0384	0.0454	0.9621	118.5959
**De Optimized**	0.0334	0.0366	0.9686	237.7129
**E-D Optimized**	0.0337	0.0371	0.9684	118.2294

**Table 6:** SOC prediction results for Type A vehicle.

	MSE	MAE	R^2^	Time/ s
**Unoptimized**	0.0476	0.0818	0.9525	238.0889
**En Optimized**	0.0484	0.0837	0.9522	117.2799
**De Optimized**	0.0406	0.0702	0.9597	238.0170
**E-D Optimized**	0.0411	0.0714	0.9594	118.5864

**Table 7:** SOC prediction results for Type B vehicle.

	MSE	MAE	R^2^	Time/ s
**Unoptimized**	0.0534	0.0941	0.9474	237.6652
**En Optimized**	0.0548	0.0974	0.9431	116.7638
**De Optimized**	0.0412	0.0822	0.9582	237.9108
**E-D Optimized**	0.0415	0.0835	0.9579	117.8201

**Table 8:** SOC prediction results for No. 2 Type A vehicle.

	MSE	MAE	R^2^	Time/ s
**Unoptimized**	0.0805	0.1768	0.9361	232.3846
**En Optimized**	0.0808	0.1772	0.9356	114.4002
**De Optimized**	0.0701	0.1664	0.9458	232.2894
**E-D Optimized**	0.0704	0.1685	0.9454	115.3439

**Table 9:** SOC prediction results for No. 4 Type B vehicle.

	MSE	MAE	R^2^	Time/ s
**Unoptimized**	0.0807	0.1722	0.9375	237.5623
**En Optimized**	0.0823	0.1741	0.9372	115.8042
**De Optimized**	0.0681	0.1464	0.9471	237.7444
**E-D Optimized**	0.0684	0.1483	0.9468	116.9470

As can be seen from Figs 4 to 11, after the En-De optimization, the prediction fitting degree of the Informer model has improved. From Tables 2–9, it can be observed that after the En-De optimization, the R^2^ indicator has improved by 15%, both MSE and MAE have decreased, and the prediction time has also been reduced by 50%. From the discussion in Section 4.2, it is known that after the En-De optimization, the prediction accuracy of the Informer model has improved by 15%, and the prediction speed has increased by 100%. Based on health assessment, optimizing the En-De module can enhance the prediction performance of the Informer model.

### 5.4 Logic optimization verification

Comparing Fig 10 and 11 with Fig 4–7, Table 8 and 9 with Table 2–7, it is easy to see that when the operation condition of the electric vehicle is changed from a single charge/discharge state to a complex operation state with multi-state switching, the prediction performance of the Informer is obviously degraded. According to Section 3.1, the prediction performance degradation is caused by the truncation error of SOC. In order to reduce the adverse effect of truncation error, it is necessary to optimize the prediction logic of Informer.

A prediction logic optimization scheme is given in Section 3.2. In order to verify whether the proposed scheme can reduce the influence of truncation error and improve the SOC prediction performance of Informer, the operation data of No. 3 Type A vehicle and No. 2 Type B vehicle in data set 4 are selected for simulation test from July to October respectively.

The monthly simulation test of each vehicle includes not only the SOC prediction effect charts before and after logic optimization, but also the prediction effect charts of motor temperature after logic optimization and the corresponding prediction result table. Prediction 1 represents the prediction effect charts before logic optimization, and Prediction 2 represents the prediction effect charts after logic optimization.

In order to avoid the over-fitting phenomenon caused by repeated training on the same data, for the same model before and after the logic optimization, the data of the same vehicle in the same month and in different time periods are selected for simulation verification.

Taking Fig 3 as an example, parameter prediction involves pre-computation and is not performed during formal predictions. On the other hand, SOC calculation is a simple arithmetic operation that does not involve the Informer model, taking less than 0.1% of the time required for temperature prediction. Therefore, after optimizing the logic, the time required for motor temperature prediction is added to that for SOC prediction. Only the SOC prediction result table includes a column for prediction time, while the motor temperature prediction table removes the prediction time column.

**Fig 12:**
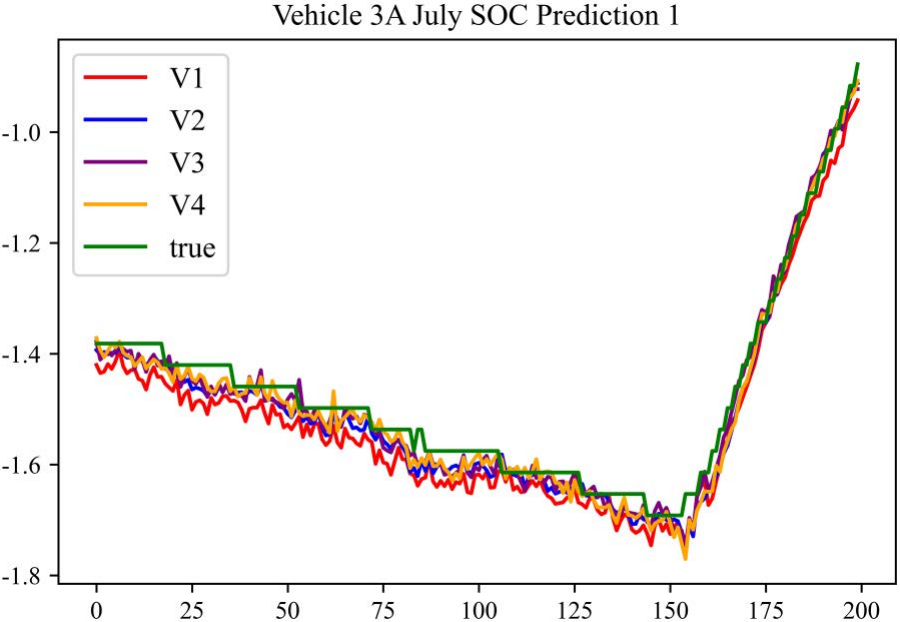
SOC prediction chart before logic optimization for 3 A-7.

**Fig 13:**
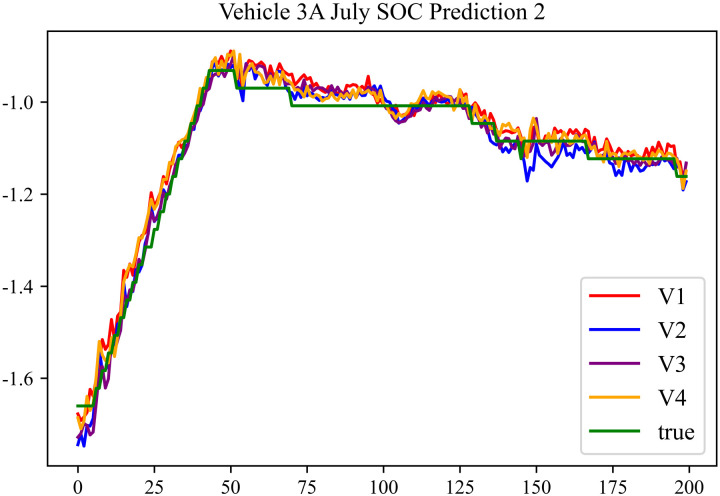
SOC prediction chart after logic optimization for 3 A-7.

**Fig 14:**
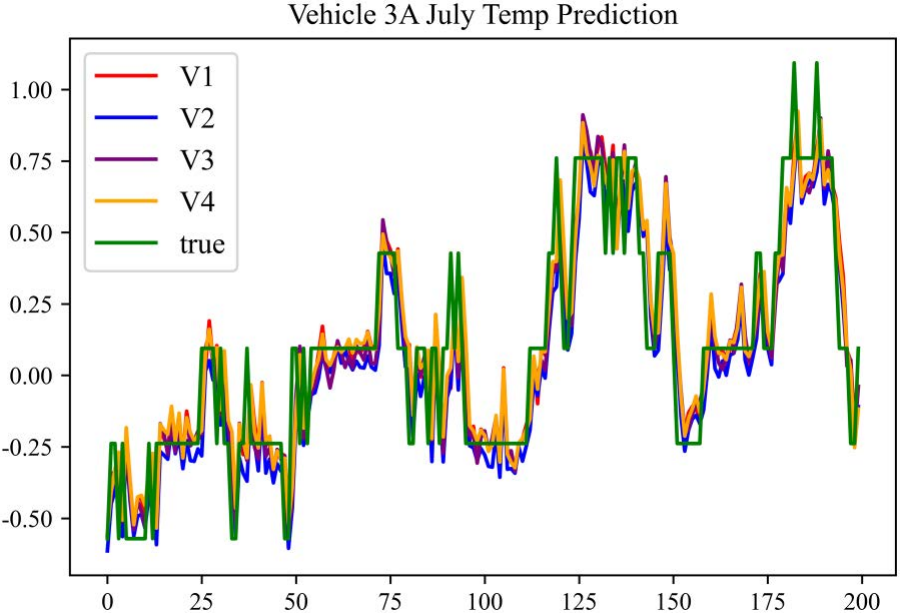
Motor temperature prediction chart for 3 A-7.

The prediction results using the July data from No. 3, Type A Vehicle are shown in Figs 12–14.

The prediction results for No. 3, Type A Vehicle for the month of July are presented in Tables 10–12.

**Table 10:** SOC prediction results before logic optimization for 3 A-7.

	MSE	MAE	R^2^	Time/ s
**Unoptimized**	0.0491	0.1846	0.9431	235.9716
**En Optimized**	0.0495	0.1877	0.9426	114.5034
**De Optimized**	0.0388	0.1442	0.9515	235.7452
**E-D Optimized**	0.0391	0.1458	0.9512	114.7039

**Table 11:** SOC prediction results after logic optimization for 3 A-7.

	MSE	MAE	R^2^	Time/ s
**Unoptimized**	0.0338	0.1338	0.9554	237.2037
**En Optimized**	0.0342	0.1352	0.9551	117.0267
**De Optimized**	0.0276	0.1204	0.9627	237.1915
**E-D Optimized**	0.0279	0.1223	0.9624	117.1969

**Table 12:** Motor temperature prediction results for 3 A-7.

	MSE	MAE	R^2^
**Unoptimized**	0.0422	0.1518	0.9491
**En Optimized**	0.0426	0.1536	0.9489
**De Optimized**	0.0339	0.1284	0.9576
**E-D Optimized**	0.0342	0.1298	0.9573

**Fig 15:**
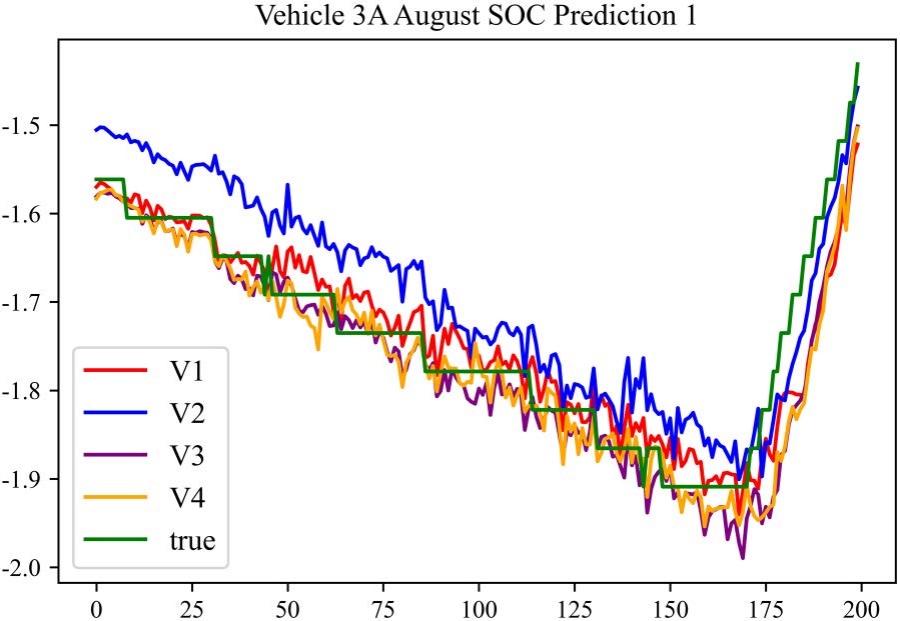
SOC prediction chart before logic optimization for 3 A-8.

**Fig 16:**
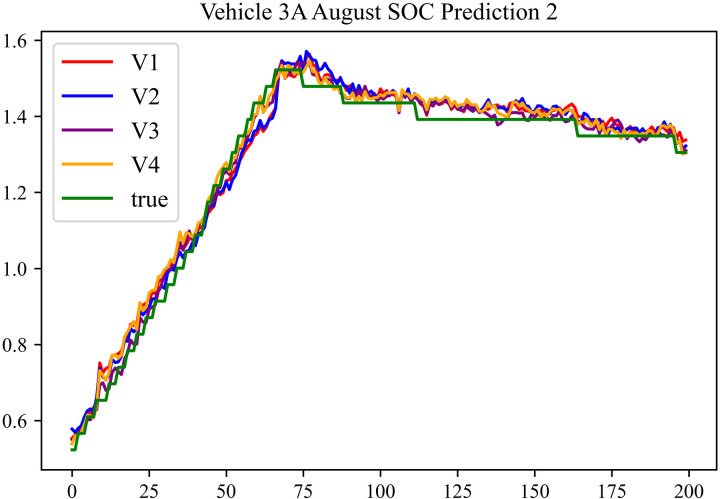
SOC prediction chart after logic optimization for 3 A-8.

**Fig 17:**
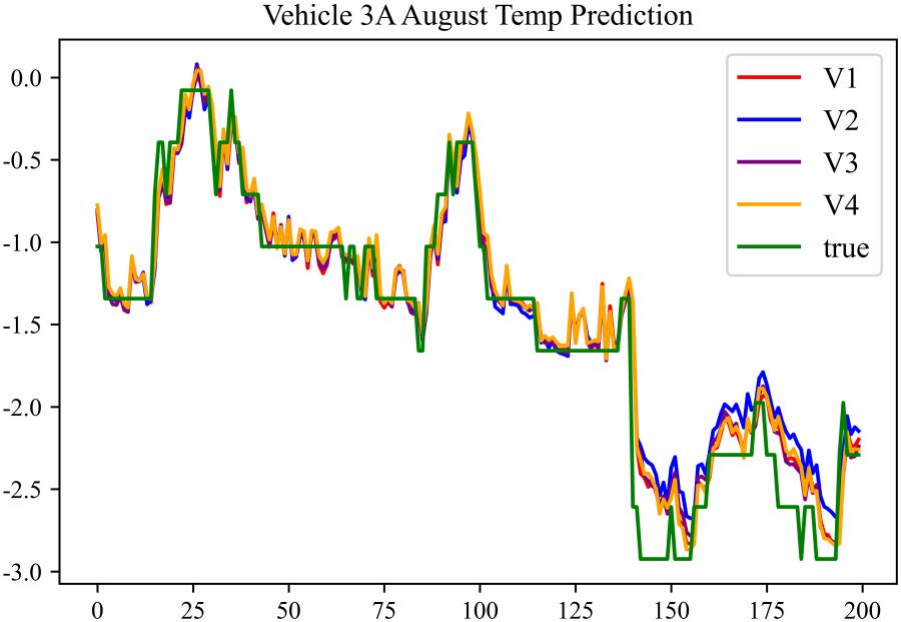
Motor temperature prediction chart for 3 A-8.

The prediction results using the August data from No. 3, Type A Vehicle are shown in Figs 15–17.

The prediction results for No. 3, Type A Vehicle for the month of August are presented in Tables 13–15.

**Table 13:** SOC prediction results before logic optimization for 3 A-8.

	MSE	MAE	R^2^	Time/ s
**Unoptimized**	0.0458	0.1776	0.9462	234.6638
**En Optimized**	0.0461	0.1793	0.9458	114.9243
**De Optimized**	0.0354	0.1373	0.9545	235.503
**E-D Optimized**	0.0358	0.1387	0.9542	114.911

**Table 14:** SOC prediction results after logic optimization for 3 A-8.

	MSE	MAE	R^2^	Time/ s
**Unoptimized**	0.0317	0.1268	0.9574	236.4098
**En Optimized**	0.0320	0.1283	0.9572	116.1468
**De Optimized**	0.0261	0.1172	0.9641	236.3545
**E-D Optimized**	0.0264	0.1188	0.9639	116.8570

**Table 15:** Motor temperature prediction results for 3 A-8.

	MSE	MAE	R^2^
**Unoptimized**	0.0344	0.1354	0.9498
**En Optimized**	0.0347	0.1378	0.9496
**De Optimized**	0.0266	0.1142	0.9579
**E-D Optimized**	0.0268	0.1153	0.9576

**Fig 18:**
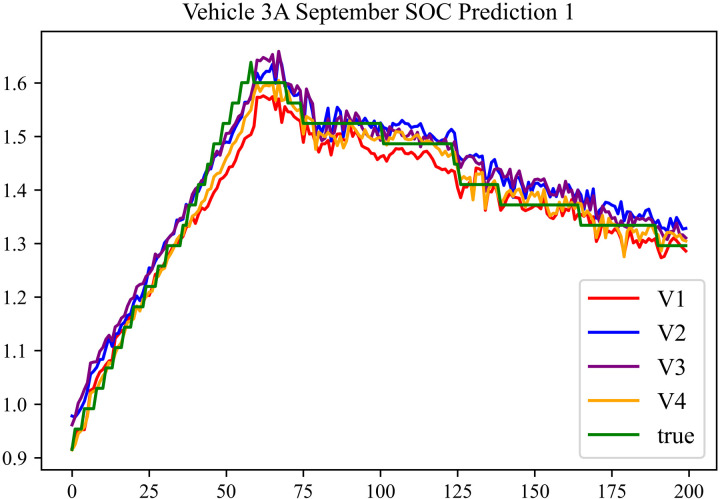
SOC prediction chart before logic optimization for 3 A-9.

**Fig 19:**
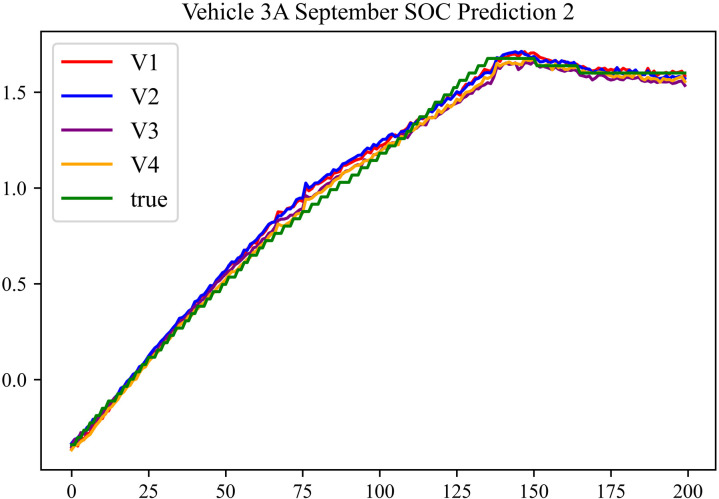
SOC prediction chart after logic optimization for 3 A-9.

**Fig 20:**
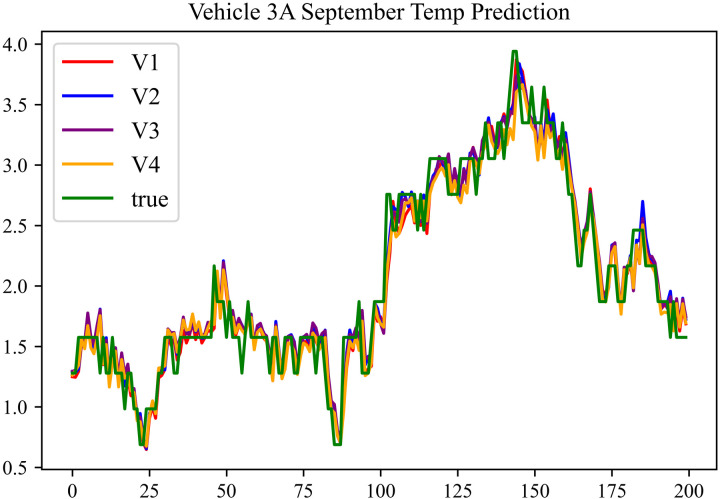
Motor temperature prediction chart for 3 A-9.

The prediction results using the September data from No. 3, Type A Vehicle are shown in Figs 18–20.

The prediction results for No. 3, Type A Vehicle for the month of September are presented in Tables 16–18.

**Table 16:** SOC prediction results before logic optimization for 3 A-9.

	MSE	MAE	R^2^	Time/ s
**Unoptimized**	0.0524	0.1903	0.9405	234.3122
**En Optimized**	0.0528	0.1926	0.9401	116.6624
**De Optimized**	0.0412	0.1516	0.9498	236.4583
**E-D Optimized**	0.0415	0.1535	0.9495	116.3426

**Table 17:** SOC prediction results after logic optimization for 3 A-9.

	MSE	MAE	R^2^	Time/ s
**Unoptimized**	0.0348	0.1322	0.9536	236.5355
**En Optimized**	0.0351	0.1347	0.9533	116.6276
**De Optimized**	0.0274	0.1175	0.9608	236.8728
**E-D Optimized**	0.0278	0.1192	0.9604	116.1632

**Table 18:** Motor temperature prediction results for 3 A-9.

	MSE	MAE	R^2^
**Unoptimized**	0.0392	0.1442	0.9357
**En Optimized**	0.0405	0.1466	0.9354
**De Optimized**	0.0348	0.1332	0.9468
**E-D Optimized**	0.0352	0.1356	0.9465

**Fig 21:**
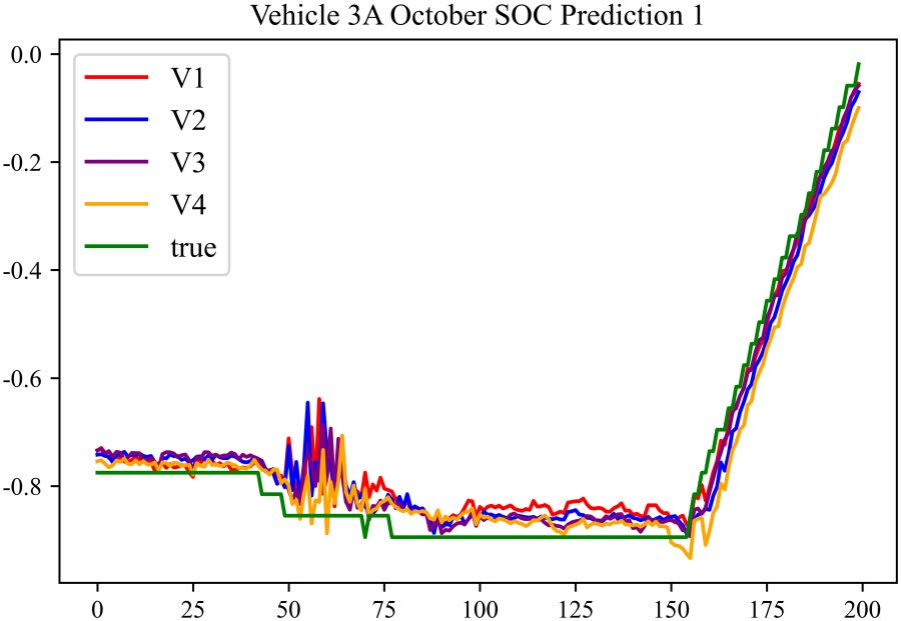
SOC prediction chart before logic optimization for 3 A-10.

**Fig 22:**
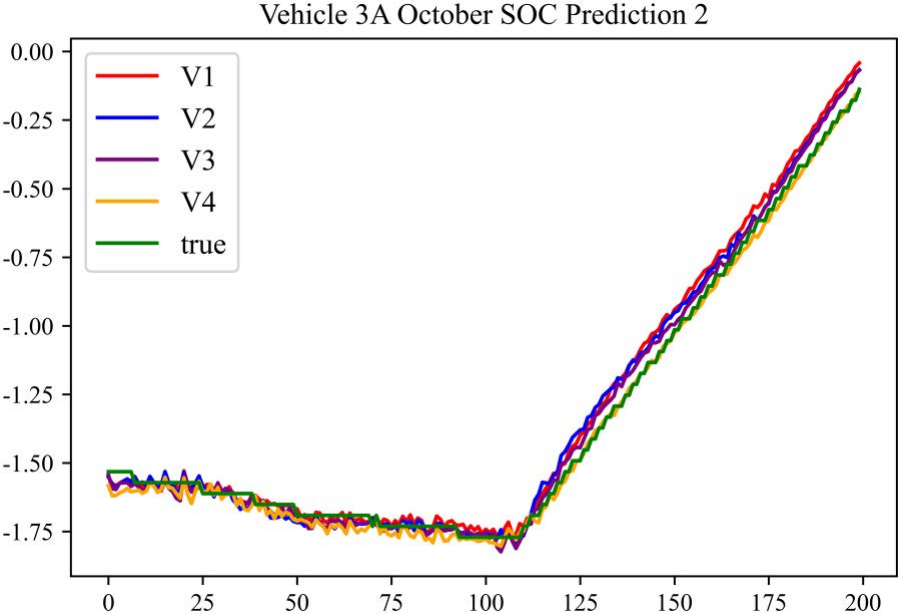
SOC prediction chart after logic optimization for 3 A-10.

**Fig 23:**
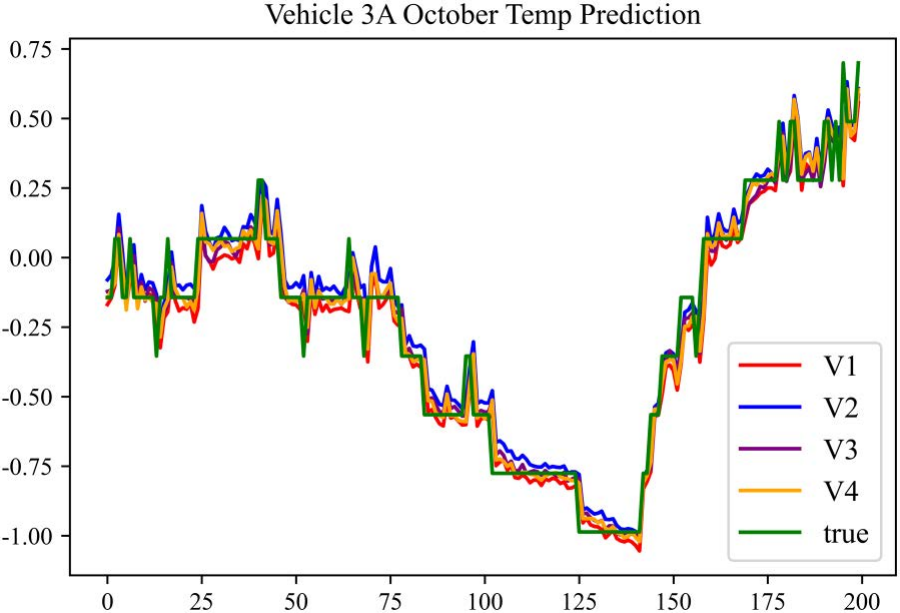
Motor temperature prediction chart for 3 A-10.

The prediction results using the October data from No. 3, Type A Vehicle are shown in Figs 21–23.

The prediction results for No. 3, Type A Vehicle for the month of October are presented in Tables 19–21.

**Table 19:** SOC prediction results before logic optimization for 3 A-10.

	MSE	MAE	R^2^	Time/ s
**Unoptimized**	0.0409	0.1542	0.9506	237.1664
**En Optimized**	0.0412	0.1573	0.9503	117.4308
**De Optimized**	0.0311	0.1302	0.9584	237.6843
**E-D Optimized**	0.0313	0.1317	0.9582	117.4185

**Table 20:** SOC prediction results after logic optimization for 3 A-10.

	MSE	MAE	R^2^	Time/ s
**Unoptimized**	0.0283	0.1254	0.9613	236.7541
**En Optimized**	0.0286	0.1274	0.9611	117.1525
**De Optimized**	0.0214	0.1103	0.9674	237.2971
**E-D Optimized**	0.0217	0.1116	0.9671	117.1877

**Table 21:** Motor temperature prediction results for 3 A-10.

	MSE	MAE	R^2^
**Unoptimized**	0.0179	0.1006	0.9697
**En Optimized**	0.0182	0.1034	0.9692
**De Optimized**	0.0154	0.0894	0.9746
**E-D Optimized**	0.0156	0.0904	0.9742

**Fig 24:**
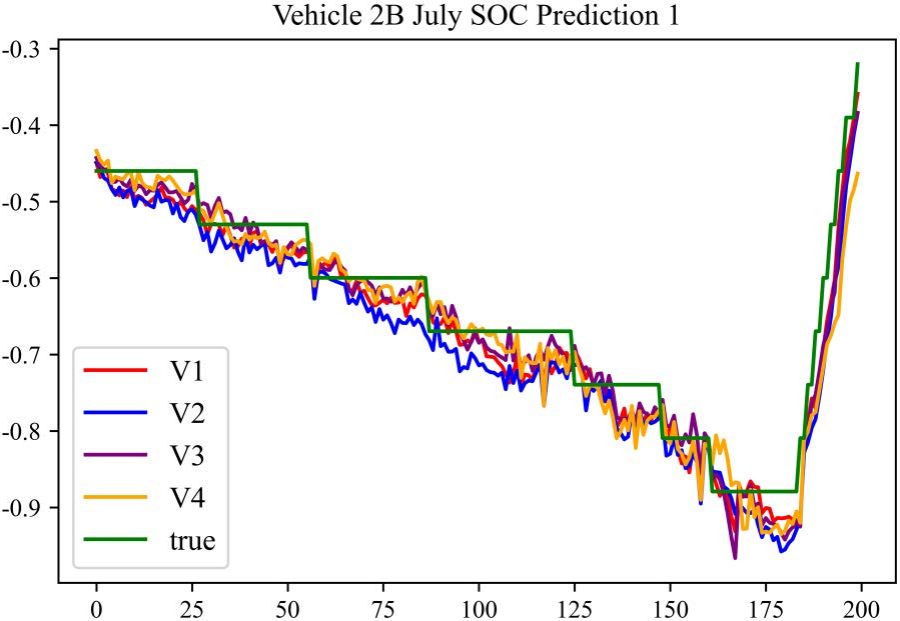
SOC prediction chart before logic optimization for 2B-7.

**Fig 25:**
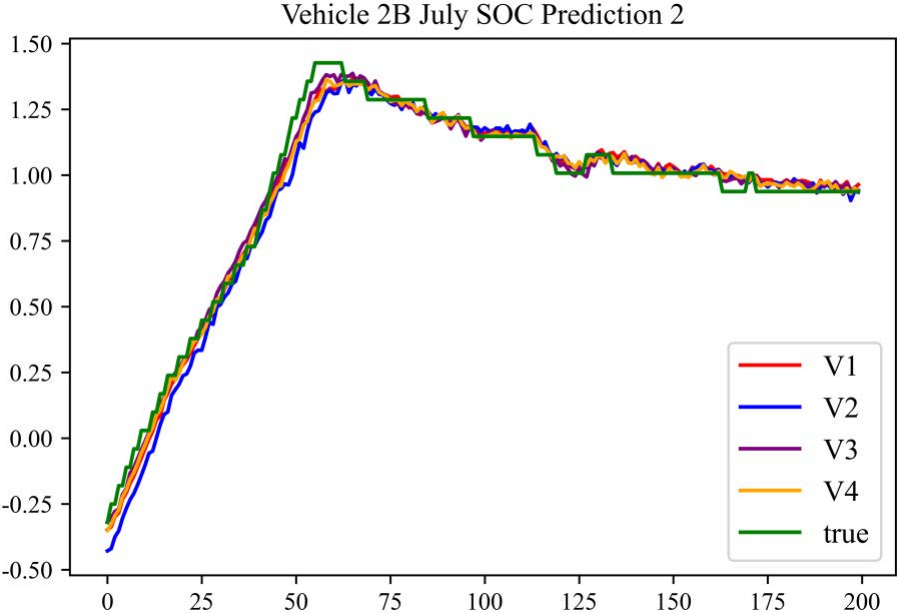
SOC prediction chart after logic optimization for 2B-7.

**Fig 26:**
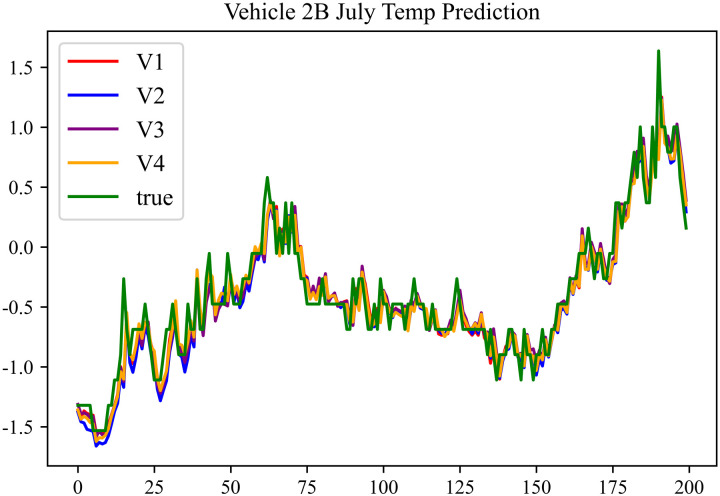
Motor temperature prediction chart for 2B-7.

The prediction results using the July data from No. 2, Type B Vehicle are shown in Figs 24–26.

The prediction results for No. 2, Type B Vehicle for the month of July are presented in Tables 22–24.

**Table 22:** SOC prediction results before logic optimization for 2B-7.

	MSE	MAE	R^2^	Time/ s
**Unoptimized**	0.0563	0.1969	0.9469	236.1494
**En Optimized**	0.0575	0.2003	0.9459	114.7602
**De Optimized**	0.0461	0.1736	0.9556	236.8764
**E-D Optimized**	0.0468	0.1764	0.9551	114.5483

**Table 23:** SOC prediction results after logic optimization for 2B-7.

	MSE	MAE	R^2^	Time/ s
**Unoptimized**	0.0439	0.1698	0.9588	237.1540
**En Optimized**	0.0448	0.1713	0.9582	116.9603
**De Optimized**	0.0374	0.1472	0.9651	237.2668
**E-D Optimized**	0.0381	0.1494	0.9645	116.2838

**Table 24:** Motor temperature prediction results for 2B-7.

	MSE	MAE	R^2^
**Unoptimized**	0.0374	0.1357	0.9634
**En Optimized**	0.0378	0.1371	0.9631
**De Optimized**	0.0309	0.1266	0.9697
**E-D Optimized**	0.0316	0.1278	0.9693

**Fig 27:**
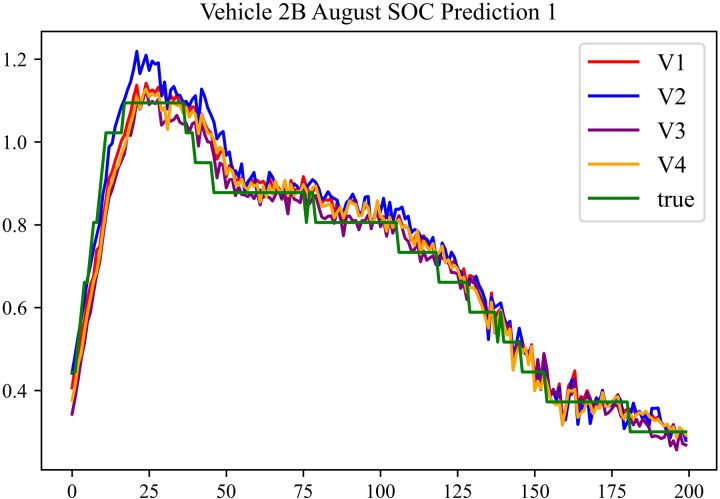
SOC prediction chart before logic optimization for 2B-8.

**Fig 28:**
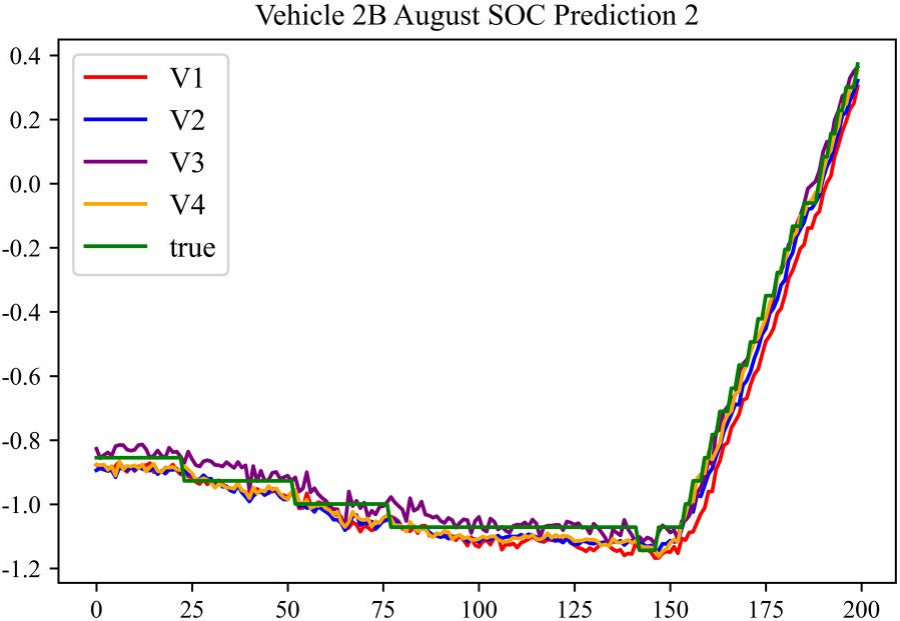
SOC prediction chart after logic optimization for 2B-8.

**Fig 29:**
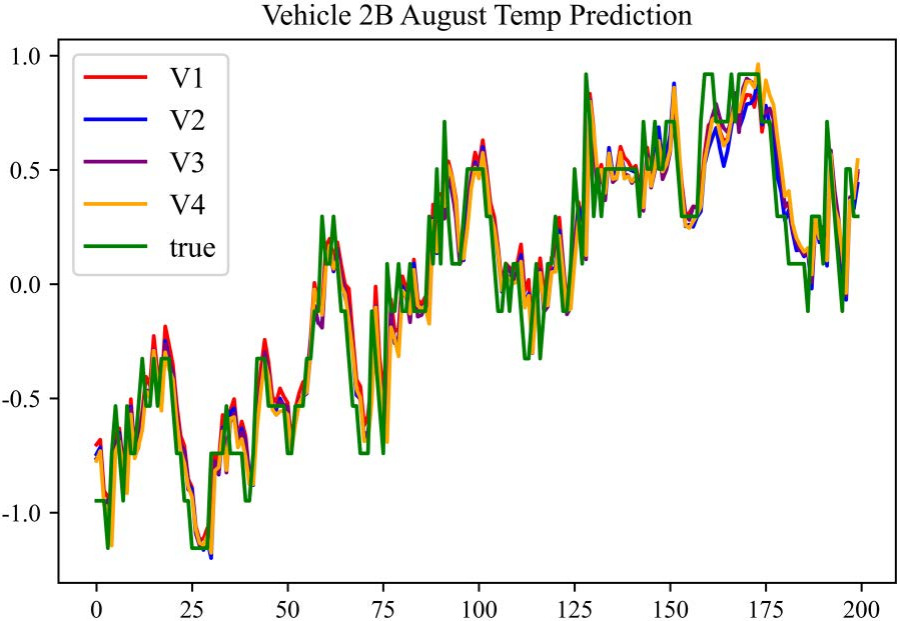
Motor temperature prediction chart for 2B-8.

The prediction results using the August data from No. 2, Type B Vehicle are shown in Figs 27–29.

The prediction results for No. 2, Type B Vehicle for the month of August are presented in Tables 25–27.

**Table 25:** SOC prediction results before logic optimization for 2B-8.

	MSE	MAE	R^2^	Time/ s
**Unoptimized**	0.0628	0.2213	0.9402	235.0011
**En Optimized**	0.0632	0.2234	0.9398	116.8813
**De Optimized**	0.0533	0.1861	0.9506	235.6224
**E-D Optimized**	0.0538	0.1875	0.9502	116.0227

**Table 26:** SOC prediction results after logic optimization for 2B-8.

	MSE	MAE	R^2^	Time/ s
**Unoptimized**	0.0489	0.1791	0.9544	237.5720
**En Optimized**	0.0496	0.1798	0.9541	116.7867
**De Optimized**	0.0406	0.1542	0.9616	236.4508
**E-D Optimized**	0.0411	0.1556	0.9612	116.7750

**Table 27:** Motor temperature prediction results for 2B-8.

	MSE	MAE	R^2^
**Unoptimized**	0.0365	0.1277	0.9459
**En Optimized**	0.0369	0.1284	0.9455
**De Optimized**	0.0317	0.1204	0.9541
**E-D Optimized**	0.0321	0.1213	0.9538

**Fig 30:**
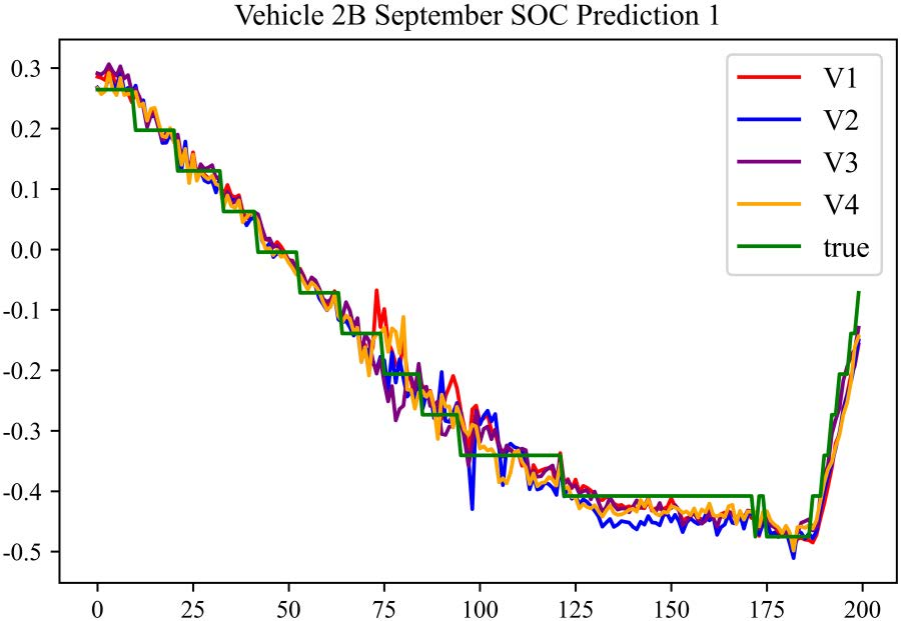
SOC prediction chart before logic optimization for 2B-9.

**Fig 31:**
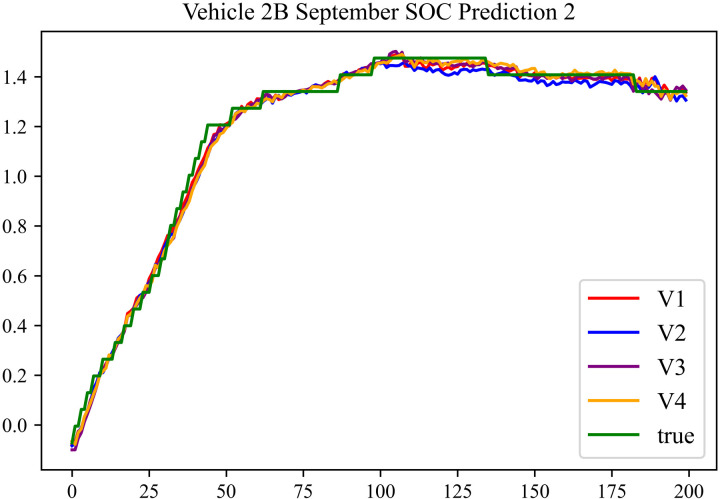
SOC prediction chart after logic optimization for 2B-9.

**Fig 32:**
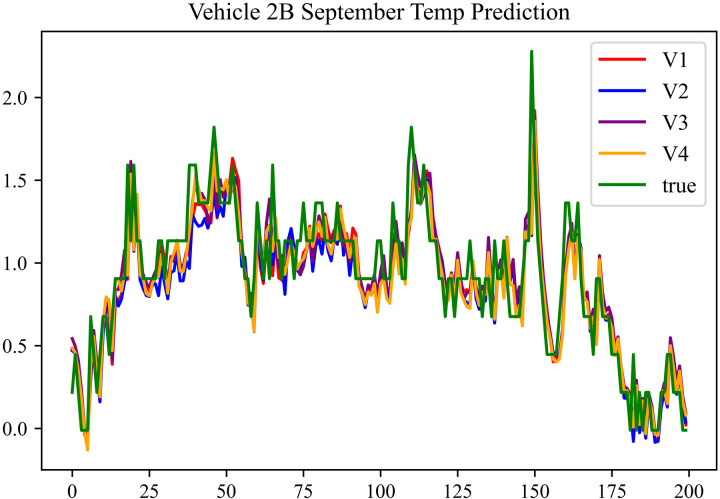
Motor temperature prediction chart for 2B-9.

The prediction results using the September data from No. 2, Type B Vehicle are shown in Figs 30–32.

The predicted results for No. 2, Type B Vehicle in September are shown in Tables 28–30.

**Table 28:** SOC prediction results for 2B-9 before logic optimization.

	MSE	MAE	R^2^	Time/ s
**Unoptimized**	0.0581	0.1932	0.9485	235.6866
**En Optimized**	0.0586	0.1954	0.9479	116.3640
**De Optimized**	0.0445	0.1706	0.9573	236.1334
**E-D Optimized**	0.0452	0.1726	0.9569	116.4136

**Table 29:** SOC prediction results for 2B-9 after logic optimization.

	MSE	MAE	R^2^	Time/ s
**Unoptimized**	0.0426	0.1671	0.9591	236.1355
**En Optimized**	0.0431	0.1682	0.9587	117.5051
**De Optimized**	0.0363	0.1487	0.9653	236.8813
**E-D Optimized**	0.0367	0.1498	0.9647	118.1003

**Table 30:** Motor temperature prediction results for 2B-9.

	MSE	MAE	R^2^
**Unoptimized**	0.0703	0.1775	0.9594
**En Optimized**	0.0711	0.1798	0.9591
**De Optimized**	0.0633	0.1564	0.9664
**E-D Optimized**	0.0638	0.1572	0.9662

**Fig 33:**
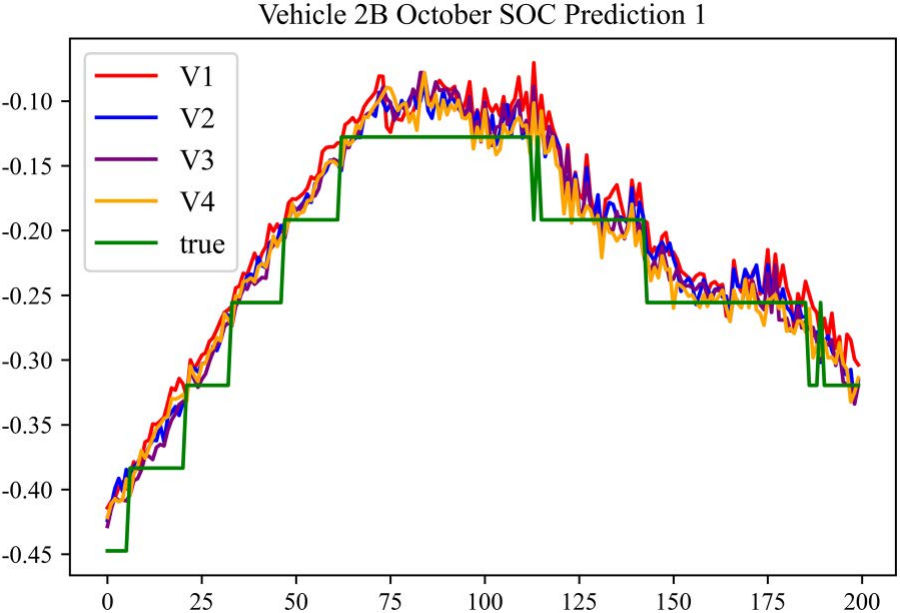
SOC prediction chart for 2B-10 before logic optimization.

**Fig 34:**
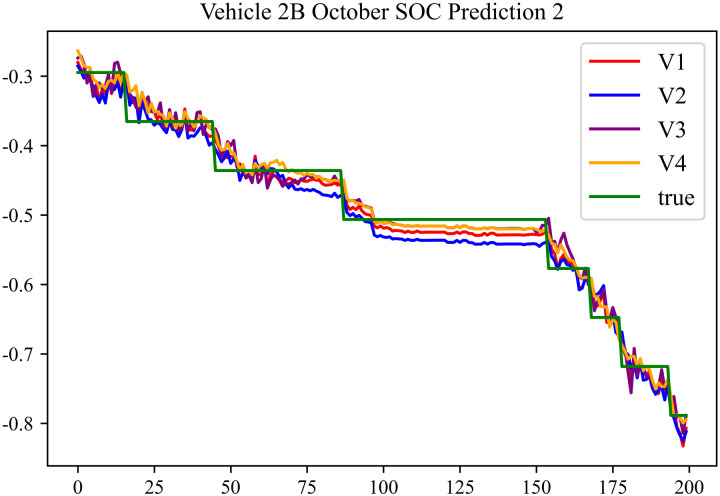
SOC prediction chart for 2B-10 after logic optimization.

**Fig 35:**
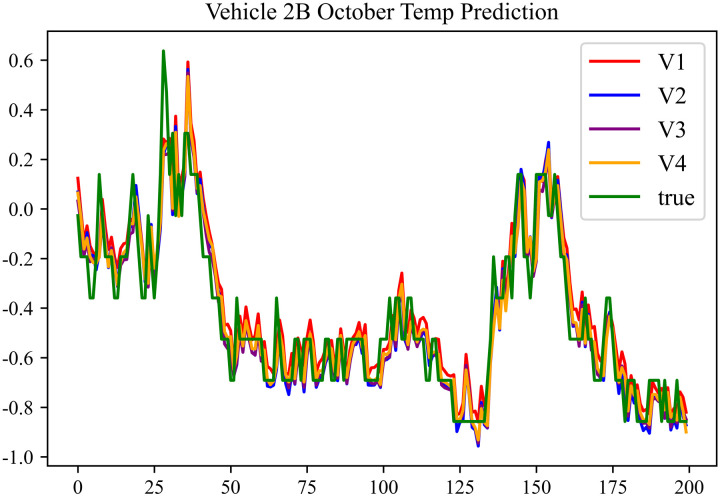
Motor temperature prediction chart for 2B-10.

The prediction effects using the October data for No. 2, Type B Vehicle are shown in Figs 33 to 35.

The prediction results for No. 2, Type B Vehicle in October are shown in Tables 31–33.

**Table 31:** SOC prediction results for 2B-10 before logic optimization.

	MSE	MAE	R^2^	Time/ s
**Unoptimized**	0.0565	0.1981	0.9472	235.2969
**En Optimized**	0.0568	0.1994	0.9469	115.5625
**De Optimized**	0.0465	0.1744	0.9554	235.1836
**E-D Optimized**	0.0471	0.1772	0.9548	115.1359

**Table 32:** SOC prediction results for 2B-10 after logic optimization.

	MSE	MAE	R^2^	Time/ s
**Unoptimized**	0.0436	0.1684	0.9590	236.5082
**En Optimized**	0.0441	0.1702	0.9586	117.2097
**De Optimized**	0.0369	0.1454	0.9654	237.0620
**E-D Optimized**	0.0376	0.1474	0.9651	116.9355

**Table 33:** Motor temperature prediction results for 2B-10.

	MSE	MAE	R^2^
**Unoptimized**	0.0294	0.1102	0.9686
**En Optimized**	0.0298	0.1121	0.9681
**De Optimized**	0.0221	0.0986	0.9736
**E-D Optimized**	0.0224	0.1013	0.9732

From Figs 13, 16, 19, 22, 25, 28, 31, and 34, it can be seen that the prediction logic optimization does not conflict with the En-De optimization. From Tables 11, 14, 17, 20, 23, 26, 29, and 32, it can be observed that after optimizing the prediction logic, the En-De optimization effect mentioned in Section 4.3, which improved prediction accuracy by 15% and prediction speed by 100%, has been retained. Furthermore, as indicated by Figs 12–35 and Tables 10–33, after optimizing the prediction logic, R^2^ metric improved by around 20%. As discussed in Section 4.2, after optimizing the prediction logic, Informer’s prediction accuracy improved by around 20%, and the precision improvement from the logic optimization coexists with the precision improvement from the En-De optimization mentioned in Section 4.3. By conducting health assessments, optimizing the prediction logic can mitigate truncation error effects and enhance the predictive performance of the Informer model.

## 6 Conclusion

Through health assessment, this study derived a health matrix. Simultaneously, improving the encoder and decoder optimizes the model structure, thereby synchronously enhancing the Informer’s prediction accuracy and speed. Building on this basis, the study indirectly predicts SOC by conducting health assessments, optimizing prediction logic, introducing basic state variables, and employing equivalent conversion to mitigate truncation error effects. This approach further enhances the Informer prediction accuracy. Through simulation test, the study validated the En-De optimization Informer model, achieving approximately a 15% increase in prediction accuracy and a 100% increase in prediction speed. After optimizing the prediction logic, the Informer model, built on the foundation of En-De optimization, reduces the impact of truncation errors, thereby further enhancing prediction accuracy by around 20%. Compared to the unoptimized Informer model, the Informer optimization based on health assessment incurs lower prediction costs and delivers superior prediction performance in SOC prediction for electric vehicles.

**Table:** 

**Nomenclature**
*Q* Weight Matrix
*R* Correlation Matrix
*A* Health Matrix
*N* Matrix Dimension
*D* Coefficient Matrix
$X^{\left(i-1\right)}$ Input of *i* - th encoding layer
$X^{\left(i\right)}$ Output of *i* - th encoding layer
*Conv* Convolutional Calculation
*Attention* Probabilistic Sparse Self-Attention
*p* Output of Self-attention sublayer
$\bar{Q}$ Probability Sparse Matrix
*softmax* Normalization Function
$X_{1}^{\left(i-1\right)}$ Optimized input of *i* - th encoding layer
$c_{0}$ Decoding initial input
$c_{L}$ Decoding output
$c_{i-1}$ Input of *i* - th decoding sublayer
$Decoder_{i}\left(\cdot \right)$ Decoding computation of *i* - th decoding sublayer
*y* Characteristic data at the prediction time
*h* Health Factor
$x_{i}$ Decoding output obtained by $Decoder_{i}\left(\cdot \right)$
$X_{de}^{t}$ Sequence form of $c_{0}$
$X_{token}^{t}$ Encoded Output
$X_{0}^{t}$ Sequence Placeholder with a Scalar of 0
*L* Sequence Length
*k* Characteristic Influence Multiplier
*m* Influence Coefficient of Motor Temperature
*n* Influence Coefficient of Other Features
Δx Amount of Change for Selected Feature
*l* Constants Related to *k*
$v_{0}$ Basic Rate of Change of SOC
$v_{1}$ SOC Prediction Rate
R^2^ Coefficient of Determination
MSE Mean Squared Error
MAE Mean Absolute Error
SOC State of Charge
SVR Support Vector Regression
GPR Gaussian Process Regression
DNN Deep Neural Network
FCEVs Fuel Cell Electric Vehicles
P&O Perturb & Observe
ANN Artificial Neural Network
En-De Encoder-Decoder
